# Determinants of social distancing adherence

**DOI:** 10.3389/fpubh.2022.977857

**Published:** 2023-01-12

**Authors:** Philip Gerretsen, Julia Kim, Eric E. Brown, Lena C. Quilty, Samantha Wells, Fernando Caravaggio, Jianmeng Song, Marcos Sanches, Branka Agic, Bruce G. Pollock, Ariel Graff-Guerrero

**Affiliations:** ^1^Centre for Addiction and Mental Health (CAMH) and Department of Psychiatry, Campbell Family Mental Health Research Institute, University of Toronto, Toronto, ON, Canada; ^2^Institute of Medical Science, University of Toronto, Toronto, ON, Canada; ^3^Institute for Mental Health Policy Research, CAMH, Toronto, ON, Canada; ^4^Dalla Lana School of Public Health, University of Toronto, Toronto, ON, Canada; ^5^Krembil Centre for Neuroinformatics, CAMH, Toronto, ON, Canada; ^6^Provincial System Support Program (PSSP), CAMH, Toronto, ON, Canada

**Keywords:** COVID-19, pandemic, social distancing adherence, disease prevention, survey, infection prevention and control, sociodemographic determinants, psychological determinants

## Abstract

**Introduction:**

Governments and public health authorities across many jurisdictions implemented social (physical) distancing measures to contain the spread of the 2019 novel coronavirus disease (COVID-19). Adherence to these measures is variable and likely influenced by various factors. This study aimed to 1) identify the individual sociodemographic, COVID-19 and social distancing related, and psychological determinants of social distancing adherence, and 2) explore regional differences in social distancing adherence in the United States (U.S.) and English-speaking Canada based on each region's discrepant response to social distancing restrictions.

**Methods:**

A web-based repeated cross-sectional survey was conducted in 4,942 English-speaking participants from the four most populous U.S. states, specifically New York, California, Texas, and Florida, and Canada (www.covid19-database.com). The study was conducted at two timepoints, from May 1 to 5, 2020 (*n* = 1,019, Canadian participants only) and from July 6 to 10, 2020 (*n* = 3,923). Separate univariate models were computed for individual sociodemographic, COVID-19 and social distancing related, and psychological determinants of social distancing adherence. To determine the total variance explained, a univariate analysis including all of the determinants was performed. Regional differences in social distancing were compared between the four U.S. states and Canada, and between the U.S. as a whole and Canada.

**Results:**

Adherence to social distancing was higher in May (mean = 4.4/5.0±0.7) compared to July (mean = 4.3/5.0±0.7) [*t*_(4940)_ = 6.96, *p* < 0.001], likely a reflection of relaxing restrictions. There were no regional differences in adherence. Sociodemographic, COVID-19 and social distancing related, and psychological determinants explained 10, 36, and 23% of the variance of social distancing adherence, respectively. Higher perceived seriousness of COVID-19 [β (SE) = 0.39 (0.01), *p* < 0.001, partial η^2^ = 0.22], lower risk propensity [β (SE) = −0.15 (0.01), *p* < 0.001, partial η^2^ = 0.06], germ aversion [β (SE) = 0.12 (0.01), *p* < 0.001, partial η^2^ = 0.03], age [β (SE) = 0.01 (0.00), *p* < 0.001, partial η^2^ = 0.02], and greater social support [β (SE) = 0.03 (0.00), *p* < 0.001, partial η^2^ = 0.02] had the largest effects on social distancing adherence.

**Conclusion:**

Public service initiatives to emphasize the serious consequences of infection and targeted interventions toward certain sociodemographic groups, such as younger adults and vulnerable individuals in greater need of social support, may help enhance the public's adherence to social distancing measures during subsequent waves of COVID-19 and future pandemics.

## Introduction

The 2019 novel coronavirus disease (COVID-19) was first identified at the end of 2019 in stallholders working at the South China Seafood Market in Wuhan, a city in the Hubei Province of China. On December 31, 2019, Chinese authorities alerted the World Health Organization (WHO) of an outbreak of a novel coronavirus. The first confirmed cases of COVID-19 were reported in the United States (U.S.) and Canada in January 2020 (1, 2). In March, the WHO characterized the COVID-19 outbreak as a pandemic. As of May 1, 2020, there were 3 million cases of COVID-19 and 224,172 deaths attributable to COVID-19 globally. Two months later, as of July 1, 2020, the global number of cases and deaths increased to 10 million and 508,055, respectively (3).

Government agencies around the world had advised social (physical) distancing and other infection prevention and control measures to prevent the transmission of COVID-19 (4, 5). These included public gathering bans, school and nonessential business closures, and advisements to maintain physical distance from non-household contacts. These interventions are considered essential to ‘flatten the curve’ (6). The aim of flattening the curve is to avoid overwhelming the healthcare system (7), as occurred in Lombardy, Italy and New York City, U.S. If enacted early, through a coordinated response among public agencies, and with cooperation of the population, mortality attributable to the pandemic can be reduced (6, 8). By pushing cases into the future, social distancing measures allow more time for the creation of additional healthcare infrastructure and the development and testing of antiviral drugs and vaccines.

There is evidence that social distancing measures have been effective in countries that enacted epidemic control measures in a timely manner (Supplementary Figure 1) (9). Prolonged or intermittent social distancing is required to mitigate further transmission of COVID-19 until the adequate dissemination of vaccines (10, 11). Lessons from past pandemics indicate that relaxing social distancing leads to an increase in cases of infection, and that the rate and number of cases is proportional to implementation delays in social distancing restrictions. Communities that enacted prolonged social distancing fared better than those that withdrew social distancing prematurely (6, 12).

Although these measures are advised by the leading health authorities around the world, including the WHO and U.S. Center for Disease Control and Prevention (CDC), other potent factors influence the political decision to maintain or relax social distancing restrictions. Specifically, the economic impact of “nonessential” business closures weighs heavily on the minds of policy decision-makers and is the rationale for loosening restrictions (13, 14). Many jurisdictions have made allowances for some businesses to be reopened and small gatherings permitted. A resurgence of cases may halt or reverse the phased relaxation of government mandated restrictions (15). Additionally, some members of society may oppose social distancing restrictions, for example, by minimizing the seriousness of COVID-19, and in turn, not adhere to infection prevention measures (16), which may undermine the public health response.

With the increase in new COVID-19 cases and deaths around the world, and given the evidence in favor of extended social distancing measures to reduce morality (6), it is important to identify the determinants of social distancing adherence. A scoping review carried out in 2021 that incorporated 84 studies investigating the determinants of social distancing adherence found that “Environmental Context and Resources” and the “Person X Environment Interaction” were the two most coded constructs identified (17). The former refers to a broad category that depicts a person's situation, such as their economic status, their demographic characteristics, the severity of the pandemic in their locality, and the specific public health policies, while the latter represents the interaction between participants' demographic characteristics or personality traits and their environment. Other frequently coded constructs include “Beliefs about Consequences,” “Emotion,” and “Social influence” (17). Another systematic review that included 28 studies about the barriers to social distancing adherence identified several individual and community level factors. Individual level factors included lacking trust in government and authority, knowledge or misconceptions about the disease, and perceived lack of threat of COVID-19 (18). Additional influences identified by this review that might hinder social distancing adherence included financial hardship, dependence on social networks and support systems, and social-cultural norms (18). Both reviews highlighted the influence of individual sociodemographic and psychological factors on adherence to social distancing restrictions.

This study aimed to add to the literature investigating the determinants of social distancing adherence. Specifically, the study intended to: (1) identify the individual sociodemographic, COVID-19 and social distancing related, and psychological determinants of social distancing adherence, and (2) explore regional differences in social distancing adherence in the U.S. and English-speaking Canada. We hypothesized a higher degree of adherence to social distancing in New York, California, and Canada compared to Florida and Texas based on each region's discrepant response to the public health recommendations at the time of the study (19).

## Methods

### Data collection

**Figure 1:**
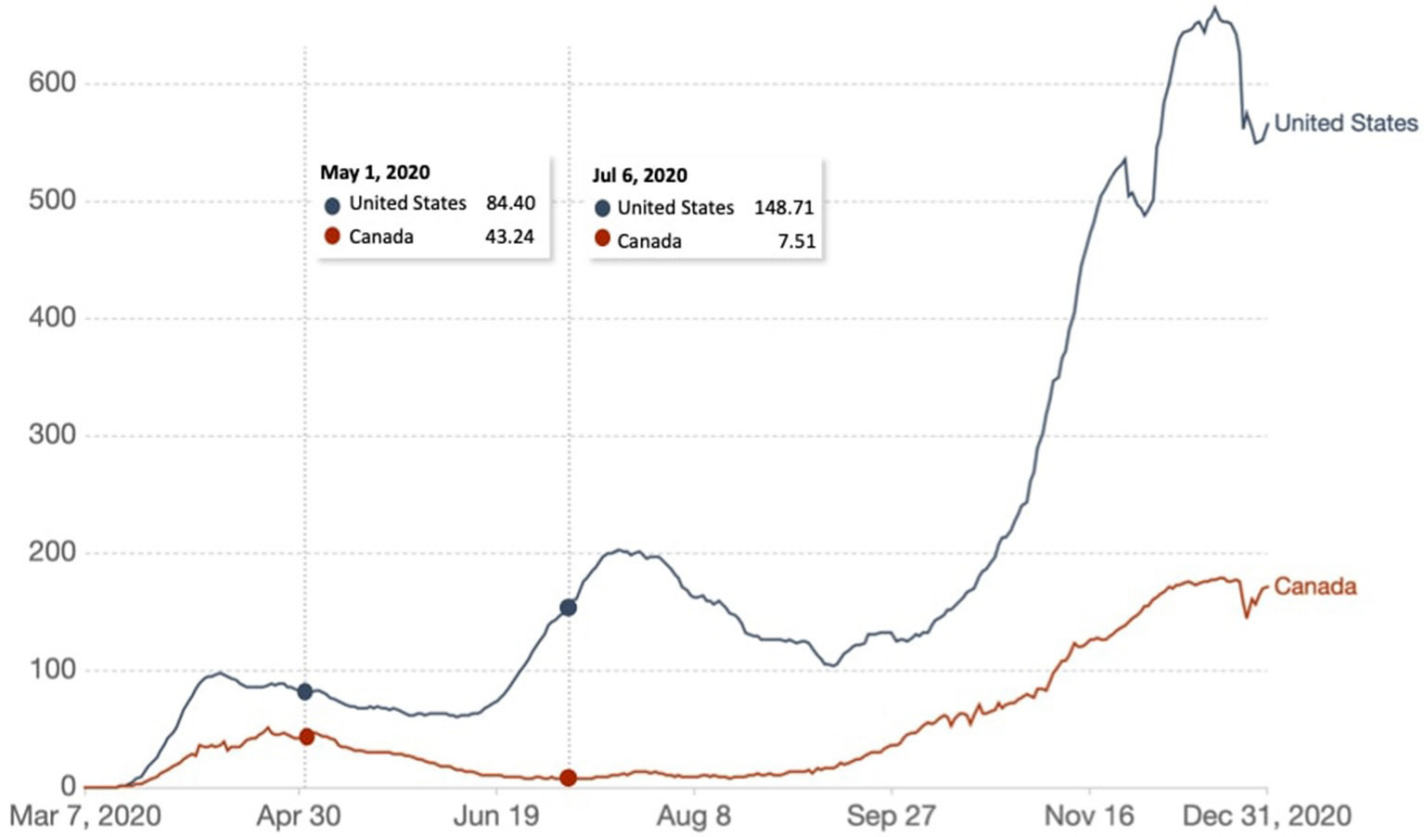
Weekly number of new confirmed COVID-19 cases per million people in the United States and Canada. The survey data was collected from May 1 to 4, 2020 (*n* = 1,019) and from July 6 to 10 (*n* = 3,923). Source: COVID-19 Data Repository by the Center for Systems Science and Engineering (CSSE) at Johns Hopkins University (20) *via* Our World in Data.

Responses from a web-based repeated cross-sectional survey were collected from 4,942 participants 18 years of age or older from the most populous U.S. states, including California, New York, Texas, and Florida, and English-speaking Canada (www.covid19-database.com). The survey was conducted from May 1 to 5 (*n* = 1,019) and from July 6 to 10, 2020 (*n* = 3,923) (Figure 1). Responses from the U.S. were collected in July only. Our target sample was quota controlled for age. All participants provided written informed consent. Information regarding survey development and quality-control can be found in Supplementary material 1. All participants provided written informed consent prior to starting the survey. The study was approved by the Centre for Addiction and Mental Health's Research Ethics Board.

### Measures

We developed the Social Distancing Adherence Scale based on recommendations from the WHO, CDC, and Public Health Agency of Canada (5, 21, 22). The scale consists of 6 items each assessed using a Likert scale, from “1, Never” to “5, Always” (Supplementary material 2). A summary score was calculated to assess the degree of social distancing adherence. The scale items had high internal consistency (Cronbach's alpha = 0.90).

Participants provided sociodemographic information and completed a battery of measures including: Citizen Trust in Government Organizations' Scale (CTGO) (23), Risk Propensity Scale (RPS) (24), Perceived Vulnerability to Disease Questionnaire (PVD) (25), Multidimensional Iowa Suggestibility Scale (MISS) (26), Duke University Religion Index (DRI), religiosity/spirituality subscale (27), Ten-Item Personality Inventory (TIPI) (28), Vaccine Attitude Examination (VAX) (29), Holistic Complementary and Alternative Medicine Questionnaire (HCAM) (30), Brief Locus-of-Control Scale (LOC) (31), General Trust Scale (GTS) (32), Authority Behavior Index (ABI) (33), Positive and Negative Affect Schedule (PANAS) (34), and Experiences in Close Relationships Scale (ECR) (35). A detailed description of each of the above measures and their internal reliability can be found in Supplementary material 3. All variables were categorized as a sociodemographic, COVID-19 and social distancing related, or psychological determinant.

### Statistical analyses

Univariate analyses were performed to identify the main determinants of social distancing adherence. A separate model was created for: (1) sociodemographic, (2) COVID-19 and social distancing related, and (3) psychological determinants. Beta (β) and partial eta squared (η^2^) values were generated and a threshold of *p* < 0.01 (0.05/3 models) was used to determine significance. Partial η^2^ values were used to define small (η^2^ = 0.01), medium (η^2^ = 0.06), and large (η^2^ = 0.14) effect sizes (36, 37). The above analyses were repeated with timepoint as a covariate (i.e., responses collected in May or July). To determine the total variance explained, a univariate analysis including all of the determinants in a single model was performed.

For exploratory purposes, the associations between the determinants and social distancing adherence were examined using spearman correlations and one-way analysis of variance (ANOVA) tests for continuous and categorical determinants, respectively. Correlation coefficients and VIF values were inspected for multicollinearity as defined by correlation coefficients ≥0.7 and VIF values ≥10.

Regional differences in social distancing adherence were compared between New York, California, Florida, Texas, and Canada, and between the U.S. as a whole and Canada using ANOVA. As no data from the U.S. was collected in May, only the responses from July were used to compare regional differences. A threshold of *p* < 0.05 was used to determine significance.

Statistical analyses were performed using SPSS Statistics (version 26 IBM Corp., Armonk, N.Y., USA).

### Subgroup analyses

Univariate analyses using the same methodology described above were performed for the following groups: males and females, and participants >60 years of age.

## Results

### Sample characteristics

Participants were broadly representative of the U.S. and Canadian population with respect to age [mean (SD) = 44.7 (17.3)] and gender (50.8% woman). The majority of participants identified as White/Caucasian (66.8%). The majority of participants identified with a religion (63.4%), with the greatest representation from Christians (45.4%), the majority identifying as Roman Catholics (20.9%). A large proportion of the sample identified as “No religion” (39.5%). The most frequently reported political affiliation was center (35.6%), followed by liberal (29.4%) and conservative (27.4%). Although most of the participants were employed (55.3%), close to 12% of participants were unemployed. Students and retirees represented 5.7 and 22.1% of the sample, respectively. The most frequently reported household income was $60,000–$99,999. The majority of the participants reported drinking alcohol (63.7%). Close to 19, 13, and 18% of the participants endorsed smoking cigarettes, using electronic cigarettes/“vape,” and cannabis products in the past week, respectively.

Participants reported knowing someone personally close who is at higher risk of COVID-19, including a healthcare worker (37.5%), someone who is elderly or has an underlying health condition (63.4%), or lives in a senior's residence (20.6%) or a long-term care home (17.4%). At the time of the survey, the majority of participants did not know anyone personally close who is or was infected with COVID-19 (71.7%). Close to 15% of the survey participants indicated that they were tested for COVID-19 and 2.6% reported that they had tested positive. Although the majority of participants believed COVID-19 originated naturally from animals to humans (64.6%), a substantial proportion believed COVID-19 originated intentionally in a lab (19.2%), accidentally in a lab (8.4%), or does not exist (1.2%) (Table 1).

**Table 1:** Participant characteristics including sociodemographic and clinical, COVID-19 and social distancing related, and psychological determinants^a^.

	**Mean (SD), Range or *N* (%)**
Social distancing adherence score	4.3 (0.7), 1.0–5.0
**Sociodemographic and clinical determinants**	
Age	44.7 (17.3)
Gender (man/woman)^b^	2,419 (49.2%) / 2,499 (50.8%)
Education (years) (*N* = 4,939)	15.2 (3.9)
Region of residence	
Canada	1,936 (39.2%)
Florida/Texas	1,004 (20.3%)
New York/California	2,002 (40.5%)
Religion (yes/no)	3,133 (66.2%) / 1,602 (33.8%)
Political affiliation	
Communism left wing or socialism	281 (5.7%)
Liberal	1,452 (29.4%)
Center	1,758 (35.6%)
Conservative	1,356 (27.4%)
Fascism right wing or authoritarianism	95 (1.9%)
Employment status	
Unemployed	595 (12.0%)
Employed	2,735 (55.3%)
Student	281 (5.7%)
Retired	1,093 (22.1%)
Household income	
< $20,000	319 (6.9%)
$20,000–$59,999	1,225 (26.4%)
$60,000–$99,999	1,364 (29.4%)
$100,000–$139,999	815 (17.6%)
$140,000 or more	918 (19.8%)
**COVID-19 and social distancing related determinants**	
Degree of social support (total score^c^) (*N* = 4,838)	13.7 (3.7), 2.0–18.0
Perceived seriousness of COVID-19 (*N* = 3,923)	4.4 (0.9), 1.0–5.0
Knowing someone personally close who	
Is a healthcare worker (yes/no)	1,852 (37.5%) / 3,090 (62.5%)
Is elderly (>60 years) or has underlying health condition (yes/no)	3,131 (63.4%) / 1,811 (36.6%)
Lives in a senior's residence (yes/no)	1,016 (20.6%) / 3,926 (79.4%)
Lives in a long-term care home (yes/no)	862 (17.4%) / 4,080 (82.6%)
Knowing someone personally close who has had COVID-19 and their outcome	
With mild symptoms	467 (9.4%)
Moderate-to-severe without hospitalization	427 (8.6%)
Moderate-to-severe with hospitalization	211 (4.3%)
Required admission to an intensive care unit	107 (2.2%)
Deceased	189 (3.8%)
Does not know anyone affected	3,541 (71.7%)
Prior laboratory testing for COVID-19 (Tested +/ Tested -/ Tested and pending/ Never tested)	128 (2.6%) / 590 (11.9%) / 37 (0.7%) / 4,187 (84.7%)
COVID-19 health risk factors (total score^d^)	0.7 (1.1), 0.0–8.0
Believing one is infected with COVID-19	1.0 (2.3), 0.0–10.0
Believing one needs testing for COVID-19	2.5 (3.2), 0.0–10.0
Reduction in income due to COVID-19	2.7 (1.5), 1.0–5.0
Negative impact of social distancing on mental health (*N* = 4,838)	2.9 (1.7), 1.0–6.0
Negative impact of COVID-19 on mental health (*N* = 4,838)	2.8 (1.6), 1,0–6.0
Origin of COVID-19	
It came about naturally likely from animals to humans	3,191 (64.6%)
It was developed intentionally in a lab	951 (19.2%)
It was made accidentally in a lab	413 (8.4%)
It doesn't really exist	58 (1.2%)
I don't know or other	329 (6.7%)
CTGO, trust in government's management of COVID-19	22.3 (8.9), 8.0–40.0
**Psychological determinants**	
RPS, Risk propensity	3.4 (1.2), 1.0–8.7
PVD, Germ aversion subscale	4.8 (1.0), 1.4–7.0
PVD, Perceived infectability subscale	3.5 (1.1), 1.0–7.0
MISS, Suggestibility	45.4 (18.0), 21.0–105.0
DRI, Religiosity/spirituality subscale	8.6 (4.1), 3.0–15.0
TIPI, Extraversion	3.8 (1.4), 1.0–7.0
TIPI, Agreeableness	4.9 (1.2), 1.0–7.0
TIPI, Conscientiousness	5.3 (1.3), 1.0–7.0
TIPI, Emotional stability	4.7 (1.3), 1.0–7.0
TIPI, Openness to experience	4.6 (1.1), 1.0–7.0
VAX, total score^e^	3.1 (1.0), 1.0–6.0
HCAM, Holistic health subscale^f^	12.1 (4.4), 5.0–30.0
HCAM, Complementary and alternative medicine subscale^f^	23.4 (4.7), 6.0–36.0
LOC, Internal	15.4 (3.3), 3.0–21.0
LOC, Chance	11.4 (4.1), 3.0–21.0
LOC, Powerful others	10.4 (4.8), 3.0–21.0
GTS, General trust	3.5 (0.8), 1.0–5.0
ABI, Attitude toward authority	77.8 (8.2), 42.0–108.0
PANAS, Positive affect score	32.3 (8.1), 10.0–50.0
PANAS, Negative affect score	20.4 (8.9), 10.0–50.0
ECR, Attachment anxiety subscale	28.9 (11.1), 8.0–56.0
ECR, Attachment avoidance subscale	29.7 (7.5), 8.0–56.0

CTGO, Citizen Trust in Government Organizations' Scale; RPS, Risk Propensity Scale; PVD, Perceived Vulnerability to Disease Questionnaire; MISS, Multidimensional Iowa Suggestibility Scale; DRI, Duke Religion/Spirituality Index; TIPI, Ten-Item Personality Inventory; VAX, Vaccination Attitudes Examination Scale; HCAM, Holistic Complementary and Alternative Medicine Questionnaire; LOC, Brief Locus-of-Control Scale; GTS, General Trust Scale; ABI, Authority Behavior Index; PANAS, Positive and Negative Affect Schedule; ECR, Experiences in Close Relationships Scale.

^a^Descriptives for race, healthcare worker status (yes/no), population density, housing situation (dwelling), marital status, substance use including alcohol, cigarettes, electronic cigarettes, and cannabis, and source of health information are included in Supplementary material 4.

b Ten participants self-identified as transgender; 10 participants as other; and 4 participants preferred not to answer or indicated that they do not know.

c A total score was derived from adding scores for the degree of satisfaction with personal relationships and support from friends.

d One point was assigned for each health risk factor (i.e., heart disease, hypertension, lung disease, diabetes, cancer, chronic kidney disease, obesity, and weakened immune system) to derive a total health risk factor score for COVID-19.

e Higher scores represent anti-vaccination attitudes.

f Higher scores represent a more negative attitude toward holistic complementary and alternative medicine.

#### Social distancing adherence

The mean (SD) social distancing adherence score was 4.3/5.0 (0.7). Adherence was higher in May [mean (SD) = 4.4/5.0 (0.7)] compared to July [mean (SD) = 4.3/5.0 (0.7) [*t*_(4940)_ = 7.0, *p* < 0.001], likely a reflection of relaxing restrictions.

There was no regional difference between New York, California, Florida, Texas, and Canada. Social distancing adherence scores were higher in the U.S. compared to Canada [mean (SD) = 4.3 (0.7) and 4.2 (0.7), respectively, F_(1, 3922)_ = 4.68, *p* = 0.031].

### Sociodemographic determinants of social distancing adherence

Sociodemographic determinants explained 10% of the variance of social distancing adherence. Sociodemographic determinants of social distancing adherence with small effects were older age, women, and left-wing political affiliation (Table 2, Supplementary Figure 2). Controlling for timepoint (i.e., responses collected in May or July) did not change the results.

**Table 2:** Univariate analysis examining the association between sociodemographic, COVID-19 and social distancing related, and psychological determinants and social distancing adherence.^1^

	**Beta**	**SE**	**t**	***p*-value**	**Partial η^2^**
**Sociodemographic and clinical determinants**					
Age	0.01	0.00	10.31	<0.001^*^	0.02^a^
Gender (man/woman^2^)	−0.13	0.02	−6.52	<0.001^*^	0.01^a^
Race					
Indigenous	0.05	0.10	0.52	0.606	0.00
Black	−0.09	0.05	−1.77	0.077	0.00
East Asian	0.11	0.04	3.06	0.002^*^	0.00
Latinx	0.16	0.04	3.88	<0.001^*^	0.00
South Asian	0.15	0.06	2.28	0.023	0.00
Other	0.03	0.04	0.74	0.459	0.00
White^2^					
Education (years)	0.01	0.00	2.04	0.041	0.00
Region of residence					
Canada	0.09	0.02	4.00	<0.001^*^	0.00
Florida/Texas	−0.01	0.03	−0.20	0.839	0.00
New York/California^2^	-	-	-	-	-
Religion (yes/no^2^)	0.04	0.02	1.91	0.057	0.00
Population density					
1,000 or less	−0.10	0.06	−1.59	0.111	0.00
1,000 to 29,999	−0.08	0.04	−2.25	0.025	0.00
30,000 to 99,999	−0.02	0.03	−0.78	0.438	0.00
100,000 or more^2^	-	-	-	-	-
Political affiliation					
Communism left wing or socialism	0.15	0.05	3.26	0.001^*^	0.00
Liberal	0.15	0.03	6.08	<0.001^*^	0.01^a^
Center^2^	-	-	-	-	-
Conservative	−0.12	0.03	−4.71	<0.001^*^	0.01^a^
Fascism right wing or authoritarianism	0.04	0.08	0.52	0.607	0.00
Healthcare worker status (yes/no^2^)	−0.03	0.03	−0.91	0.362	0.00
Employment status					
Unemployed	0.05	0.03	1.45	0.148	0.00
Employed^2^	-	-	-	-	-
Student	0.10	0.05	2.09	0.037	0.00
Retired	0.07	0.03	1.98	0.048	0.00
Dwelling					
House with a backyard^2^	-	-	-	-	-
House without a backyard	−0.02	0.06	−0.30	0.767	0.00
Apartment/condominium/loft with no or small	−0.06	0.03	−2.44	0.015	0.00
private outdoor space					
Apartment/condominium/loft with large	0.02	0.04	0.51	0.610	0.00
outdoor space					
Senior's residence	0.03	0.15	0.19	0.852	0.00
Long-term facility or nursing home	0.30	0.31	0.97	0.334	0.00
Household income					
< $20,000	−0.13	0.05	−2.67	0.008^*^	0.00
$20,000–$59,999	−0.04	0.03	−1.47	0.141	0.00
$60,000–$99,999^2^	-	-	-	-	-
$100,000–$139,999	−0.05	0.03	−1.44	0.150	0.00
$140,000 or more	0.02	0.03	0.68	0.498	0.00
Marital status (single/married^2^)	0.01	0.02	0.37	0.710	0.00
Number of persons in a household	0.02	0.01	2.60	0.009^*^	0.00
Substance use in the past week					
Alcohol use (yes/no^2^)	−0.03	0.02	−1.55	0.120	0.00
Cigarette use (yes/no^2^)	−0.03	0.03	−0.91	0.364	0.00
Electronic cigarette use (yes/no^2^)	−0.14	0.04	−3.39	0.001^*^	0.00
Cannabis use (yes/no^2^)	0.02	0.03	0.55	0.582	0.00
**COVID-19 and social distancing related determinants**					
Degree of social support (total score^3^)	0.03	0.00	9.07	0.000	0.02^a^
Perceived seriousness of COVID-19	0.39	0.01	33.23	0.000	0.22^c^
Knowing someone personally close who					
Is a healthcare worker (yes/no^2^)	−0.02	0.02	−0.69	0.490	0.00
Is elderly (>60 years) or has underlying health condition (yes/no^2^)	0.06	0.02	2.55	0.011	0.00
Lives in a senior's residence (yes/no^2^)	0.00	0.03	−0.01	0.989	0.00
Lives in a long-term care home (yes/no^2^)	−0.06	0.03	−1.78	0.075	0.00
Knowing someone personally close who has had COVID-19 and their outcome					
With mild symptoms	−0.06	0.03	−1.76	0.078	0.00
Moderate-to-severe without hospitalization	−0.04	0.03	−1.08	0.281	0.00
Moderate-to-severe with hospitalization	−0.04	0.05	−0.81	0.421	0.00
Required admission to an intensive care unit	0.00	0.07	−0.06	0.951	0.00
Deceased	−0.07	0.05	−1.37	0.172	0.00
Does not know anyone affected^2^	-	-	-	-	-
Prior laboratory testing for COVID-19					
Tested +	−0.05	0.07	−0.68	0.497	0.00
Tested -	0.01	0.03	0.18	0.858	0.00
Tested and pending	0.07	0.11	0.64	0.524	0.00
Never tested^2^	-	-	-	-	-
COVID-19 health risk factors (total score^4^)	0.01	0.01	0.83	0.405	0.00
Believing one is infected with COVID-19	−0.02	0.01	−3.69	0.000	0.00
Believing one need testing for COVID-19	0.00	0.00	−0.08	0.936	0.00
Reduction in income due to COVID-19	0.02	0.01	2.14	0.033	0.00
Negative impact of social distancing on mental health	0.03	0.01	2.42	0.015	0.00
Negative impact of COVID-19 on mental health	−0.04	0.01	−3.75	0.000	0.00
Source of health information					
Friends or family	−0.14	0.05	−2.88	0.004	0.00
Doctor	−0.04	0.03	−1.42	0.156	0.00
Social media	−0.14	0.04	−3.11	0.002	0.00
Internet	−0.04	0.03	−1.54	0.125	0.00
Radio/Podcast	−0.09	0.06	−1.34	0.179	0.00
Newspaper	−0.04	0.05	−0.95	0.342	0.00
Magazines	−0.17	0.18	−0.98	0.327	0.00
Television^2^	-	-	-	-	-
Origin of COVID-19					
It was developed intentionally in a lab	−0.04	0.03	−1.38	0.167	0.00
It was made accidentally in a lab	0.02	0.04	0.68	0.500	0.00
It doesn't really exist	−0.40	0.09	−4.28	0.000	0.01^a^
It came about naturally likely from animals to	-	-	-	-	-
humans^2^					
CTGO, trust in government's management of	0.00	0.00	−0.44	0.659	0.00
COVID-19					
**Psychological determinants**					
RPS, Risk propensity	−0.15	0.01	−17.34	<0.001^*^	0.06^b^
PVD, Germ aversion subscale	0.12	0.01	11.33	<0.001^*^	0.03^a^
PVD, Perceived infectability subscale	0.03	0.01	3.08	0.002^*^	0.00
MISS, Suggestibility	0.00	0.00	−2.87	0.004^*^	0.00
DRI, Religiosity/spirituality subscale	0.00	0.00	−1.09	0.274	0.00
TIPI, Extraversion	−0.01	0.01	−1.25	0.212	0.00
TIPI, Agreeableness	0.02	0.01	2.44	0.015	0.00
TIPI, Conscientiousness	0.01	0.01	1.10	0.273	0.00
TIPI, Emotional stability	0.01	0.01	1.05	0.293	0.00
TIPI, Openness to experience	0.06	0.01	6.72	<0.001^*^	0.01^a^
VAX, total score^5^	−0.09	0.01	−8.27	<0.001^*^	0.01^a^
HCAM, Holistic health subscale^6^	−0.01	0.00	−5.52	<0.001^*^	0.01^a^
HCAM, Complementary and alternative medicine subscale^6^	0.01	0.00	2.11	0.035	0.00
LOC, Internal	0.02	0.00	5.07	<0.001^*^	0.01^a^
LOC, Chance	0.01	0.00	2.42	0.016	0.00
LOC, Powerful others	0.00	0.00	0.93	0.355	0.00
GTS, General trust	0.10	0.01	6.98	<0.001^*^	0.01^a^
ABI, Attitude toward authority	0.00	0.00	0.67	0.503	0.00
PANAS, Positive affect score	0.01	0.00	3.43	0.001^*^	0.00
PANAS, Negative affect score	0.00	0.00	−0.86	0.390	0.00
ECR, Attachment anxiety subscale	0.00	0.00	0.01	0.996	0.00
ECR, Attachment avoidance subscale	0.01	0.00	3.83	<0.001^*^	0.00

CTGO, Citizen Trust in Government Organizations' Scale. RPS, Risk Propensity Scale; PVD, Perceived Vulnerability to Disease Questionnaire; MISS, Multidimensional Iowa Suggestibility Scale; DRI, Duke Religion/Spirituality Index; TIPI, Ten-Item Personality Inventory; HCAM, Holistic Complementary and Alternative Medicine Questionnaire; LOC, Brief Locus-of-Control Scale; GTS, General Trust Scale; ABI, Authority Behavior Index; PANAS, Positive and Negative Affect Schedule; ECR, Experiences in Close Relationships Scale.

1 A separate univariate analysis was conducted for sociodemographic, COVID-19 and social distancing, and psychological determinants. Total adjusted R^2^ for sociodemographic determinants: 0.10; COVID-19 and social distancing determinants: 0.33; psychological determinants: 0.26.

2 Reference variable.

3 A total score was derived from adding scores for the degree of satisfaction with personal relationships and support from friends.

4 One point was assigned for each health risk factor (i.e., heart disease, hypertension, lung disease, diabetes, cancer, chronic kidney disease, obesity, and weakened immune system) to derive a total health risk factor score for COVID-19.

5 Higher scores represent anti-vaccination attitudes.

6 Higher scores represent a more negative attitude toward holistic complementary and alternative medicine.

a Small effect (η^2^ = 0.01);

b Medium effect (η^2^ = 0.06);

c Large effect (η^2^ = 0.14);

* p < 0.01 (0.05/3 univariate models).

### COVID-19 and social distancing related determinants of social distancing adherence

COVID-19 and social distancing related determinants explained 33% of the variance in social distancing adherence. The main COVID-19 and social distancing related determinant with a large effect was higher perceived seriousness of COVID-19. Greater social support and believing that COVID-19 originated naturally rather than believing that it does not exist had small effects on social distancing adherence (Table 2). Controlling for timepoint did not change the results.

### Psychological determinants of social distancing adherence

Psychological determinants explained 26% of the variance in social distancing adherence. The main psychological determinant of social distancing adherence with a medium effect was lower risk propensity. Other psychological determinants with small effects were germ aversion, the personality trait of openness to experience, positive attitudes toward vaccinations and holistic health approaches, higher internal locus-of-control, and general trust in others (Table 2). Controlling for timepoint did not change the results.

**Figure 2:**
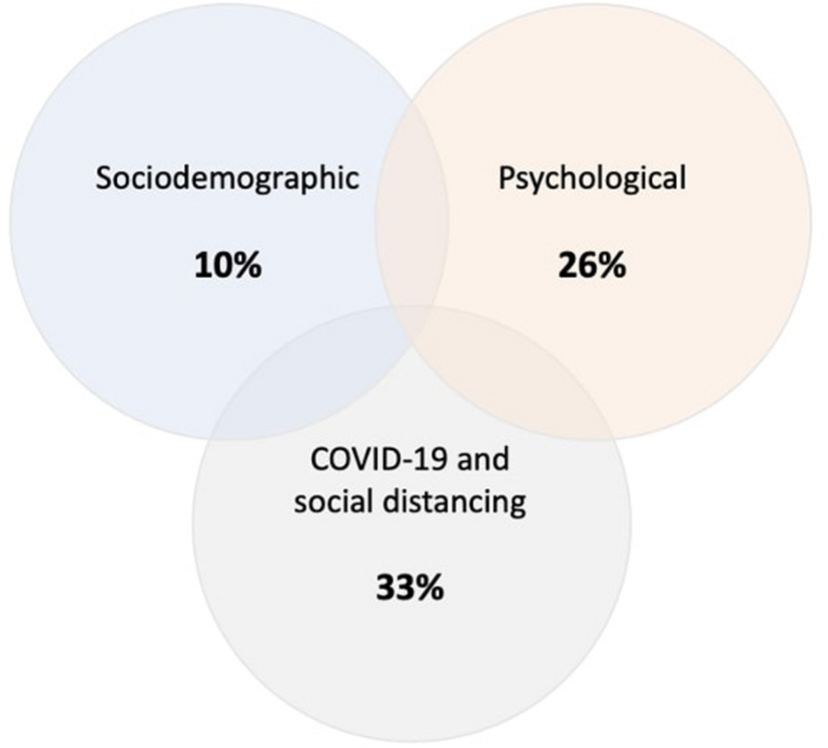
Variance of social distancing adherence explained by sociodemographic, COVID-19 and social distancing related, and psychological determinants. The percentages represent R^2^ that was derived from separate univariate models for each category. The total variance explained by sociodemographic, COVID-19 and social distancing related, and psychological determinants was 40%.

The total variance explained by sociodemographic, COVID-19 and social distancing related, and psychological determinants was 40% [F_(96, 3861)_ = 27.58, *p* < 0.001] (Figure 2).

### Exploratory analyses

Exploratory analyses examining the associations between the individual determinants and social distancing adherence are presented in Supplementary material 5.

### Subgroup analyses

Descriptive analyses and results of univariate analyses in men, women, and participants 60 years of age or older can be found in Supplementary materials 4–14.

The principal determinants of social distancing adherence identified in the subgroup analyses are consistent with those found in the main analysis. Of note, in women, less negative mental health impact of COVID-19 and source of health information (i.e., preference for television over social media) had a small effect on social distancing adherence. In men, knowing someone personally close who is elderly was associated with a small effect on social distancing adherence. Also, in men, and in participants 60 years of age or older, an avoidant attachment style emerged as a determinant of social distancing adherence with a small effect.

## Discussion

At the time of this study, perceptions of COVID-19 and the determinants of adherence to the recommended social distancing measures remained largely unknown. With the number of new cases of COVID-19 rising around much of the world, adherence with social distancing restrictions remained an active issue in relation to the containment and reduction of mortality attributable to COVID-19. While sustained social distancing strategies can save lives (6), prolonged social distancing may have considerable negative consequences, including loneliness, adverse mental health effects (38), and substantial social, educational, and economic disruption.

Our study found that adults in the U.S. and Canada were generally adherent to social distancing measures. At the time of the survey, messaging from the Government of the United States and Canada was to ‘Reopen’ (14, 39). As a whole, the U.S. states studied were modestly more adherent to social distancing restrictions than Canada. This may be due to reversal of reopening plans at that time due to the rising number of COVID-19 cases in some U.S. states, including California (see Figure 1, Timepoint 2, when regional differences were analyzed) (40).

Sociodemographic and psychological determinants explained 10% and 26% of the variance in social distancing adherence, respectively. COVID-19 and social distancing related factors explained 33% of the variance in social distancing adherence (Figure 2). The main determinant of social distancing adherence was higher perceived seriousness of COVID-19, followed by higher risk propensity. The principal finding that an individual's perception of the of seriousness of COVID-19 is consistent with the results of a systematic review that reported an individual's perception of COIVD-19 as a threat contributes to adherence to social distancing restrictions (18). Risk propensity refers to an individual's general tendency to take risks (24). Few studies have explored the role of risk propensity on social distancing behavior during COVID-19. All of these investigations, however, indicate that individuals with lower risk tolerance are more likely to adhere to social distancing restrictions, independent of the perceived seriousness or objective threat of COVID-19 (41). In contrary, people with higher risk propensity are more likely to engage in behaviors that are considered risky in the context of COVID-19 (42, 43).

In summary, our results describe individuals most likely to be nonadherent with social distancing restrictions as younger men with a right-wing political affiliation. They do not believe COVID-19 is serious or that it exists. They have a higher propensity for risk, negative attitudes toward vaccinations or holistic health approaches, a weak sense of self-agency (i.e., low internal locus of control), and are generally distrusting of others. Although there were minor differences in the determinants of social distancing adherence in men, women, and participants 60 years of age or older, the main determinants of social distancing adherence identified in these subgroups were consistent with those found in the main analysis. Other studies in varied countries have also supported our findings that age, gender, political affiliation, distrust, and perceived self-control are individual determinants that contribute to adherence to social distancing measures (44–48).

Of note, other studies have found that COVID-19 awareness of the COVID-19 pandemic and lack of concrete knowledge about the disease influence social distancing adherence, highlighting the importance of public education (18, 48, 49).

The results of our study are limited by the known biases associated with research participation, namely, individuals that consent to participate in research are often more conscientious and willing to sacrifice their time to support the greater good than are nonparticipants (50). Another limitation that is intrinsic to web-based surveys is that participants who are unfamiliar with using a computer or have no internet access are not represented. However, given the time sensitivity of the study, a web-based survey allowed for reaching a larger number participants within a short period of time without compromising validity and reliability (51). Further, we are unable to comment on the direction of the associations given the cross-sectional nature of the study.

## Conclusions

The success of public health interventions, such as social distancing, depend on public support and adherence (6). Our study identified individual sociodemographic, COVID-19 and social distancing related, and psychological determinants that can inform public health and other authorities to develop public service interventions to improve social distancing adherence and contain the spread of COVID-19 and future infections more effectively. These may include public service initiatives to emphasize the seriousness of COVID-19 and future infectious diseases, and tackle false or misleading information about them. Targeted interventions toward certain sociodemographic groups, such as younger men and vulnerable individuals in greater need of social support, and health communications promoting a sense of control over COVID-19 and future infections and their consequences may also be beneficial.

## Targeted recommendations

1) *Seriousness of infection:* Emphasize the seriousness of COVID-19, including increasing awareness of the risk of transmission, likelihood of serious illness, and the associated morbidity.

2) *Risk propensity and germ aversion:* Increase knowledge of the risk of transmission without infection prevention measures, including social distancing, and the elevated risk of mortality, particularly in the elderly. Influence perceptions by emphasizing the likelihood of a serious negative outcome with COVID-19 infection. Individuals may minimize the seriousness of COVID-19 after acquiring personal knowledge of individuals with mild cases of the infection.

3) *Social support:* Promote virtual social connection and support to address social isolation. Concerned, consistent, accessible others may alleviate one's sense of social isolation and attachment anxiety (52).

4) *Attitudes toward vaccinations:* Enhance the public's confidence in safety and effectiveness of vaccines and the systems recommending and providing it. Increase awareness that vaccination is required to prevent infection and transmission of COVID-19, and that the benefits of any safe and effective vaccine outweigh the possible consequences.

5) *Perception of holistic health:* Promote a holistic attitude where individuals are mindful of the effects of emotional wellbeing on physical health, i.e. “Mental health is health” (53).

6) *Internal locus-of-control:* Promote individual agency or sense of control over COVID-19 and its consequences (e.g., the message “Conquering COVID-19 is in my hands! By adopting good hygiene and social distancing practices, I am keeping myself, family, friends, and my community safe,” may instill a sense of control over the impact of COVID-19 and enhance one's ability to practice protective behavior).

## Data availability statement

The raw data supporting the conclusions of this article are available, without undue reservation, at: http://www.covid19-database.com/.

## Ethics statement

All studies involving human participants are reviewed and approved by the Centre for Addiction and Mental Health. Participants provided their written informed consent to participate in this study.

## Author contributions

PG, JK, and AG-G: agreement to be accountable for all aspects of the work in ensuring that questions related to the accuracy or integrity of any part of the work are appropriately investigated and resolved. All authors: substantial contributions to the conception or design of the work and/or the acquisition, analysis, or interpretation of data for the work, drafting of the manuscript and/or revising it critically for important intellectual content, final approval of the version to be published.
