# Supplementary material for: Determinants of social distancing adherence
Source: Front Public Health. 2023 Jan 12;10:977857. doi: 10.3389/fpubh.2022.977857 (PMC9879577; doi:10.3389/fpubh.2022.977857)
Supplement: Supplementary file 1 [file Data_Sheet_1.docx]

Supplementary Material

**Supplemental Material 1.** Survey development.

**Supplemental Material 2.** Social distancing adherence scale.

**Supplemental Material 3.** List of measures.

**Supplemental Material 4.** Sociodemographic, COVID-19 and social distancing related, and psychological characteristics in men, women, and participants 60 years of age or older.

**Supplemental Material 5.** Associations between sociodemographic, COVID-19 and social distancing related, and psychological determinants and social distancing adherence score in men, women, and participants 60 years of age or older.

**Supplemental Material 6.** Univariate analysis examining the association between sociodemographic determinants and social distancing adherence in men.

**Supplemental Material 7.** Univariate analysis examining the association between COVID-19 and social distancing determinants and social distancing adherence in men.

**Supplemental Material 8.** Univariate analysis examining the association between psychological determinants and social distancing adherence in men.

**Supplemental Material 9.** Univariate analysis examining the association between sociodemographic determinants and social distancing adherence in women.

**Supplemental Material 10.** Univariate analysis examining the association between COVID-19 and social distancing determinants and social distancing adherence in women.

**Supplemental Material 11.** Univariate analysis examining the association between psychological determinants and social distancing adherence in women.

**Supplemental Material 12.** Univariate analysis examining the association between sociodemographic determinants and social distancing adherence in participants 60 years of age or older.

**Supplemental Material 13.** Univariate analysis examining the association between COVID-19 and social distancing determinants and social distancing adherence in participants 60 years of age or older.

**Supplemental Material 14.** Univariate analysis examining the association between psychological determinants and social distancing adherence in participants 60 years of age or older.

**Supplemental Figure 1.** Weekly number of cases per million people prior to the second wave. Comparing the United States and Canada, who enacted social distancing restrictions relatively late and early, respectively, to Italy who were unable to or chose not to adopt social distancing restrictions early.

**Supplemental Figure 2.** Mean social distancing adherence scores across political affiliations.

**Supplemental Material 1.** Survey development

***Development and pre-testing***

The survey was tested by our team (PG, JK, and AG) and by Dynata prior to the soft launch to ensure usability and technical functionality. The soft launch data were inspected by Dynata and our team prior to the hard launch on May 4, 2020 and June 6, 2020.

***Recruitment process and survey administration***

Participants were invited by e-mail based on their demographic profile. Participants were also able to take the survey by accessing Dynata’s survey platform. Participants received panel credit points for their time and participation.

Where appropriate, the survey items were randomized to prevent bias. In order to reduce number and complexity of the questions, certain questions were asked conditionally. A maximum of one questionnaire was asked per screen. The number of questionnaire items per page ranged from 1 to a maximum of 24 questions (i.e., Authority Behavior Index (1)). The survey questions were distributed across 97 screens. Selection of a single response option was enforced throughout the survey. Participants were unable go back to previous screens and change their answers.

***Quality control***

Comprehensive IP address, digital fingerprint, and de-duplicating checks were in place to prevent the same respondents from completing the study more than once. Participants who acquired a “fixed” survey status via study termination, exceeding the quota, or completion, could not access the survey again. Data were inspected by Dynata as part of standard quality control procedures, including open end verbatim and racer checks. Open end verbatim checks were done manually to identify and replace key smash or poor-quality inputs. The racer check was set at 30% of the median survey length, and those who completed the survey in less than 10 minutes were removed. Only completed questionnaires were considered.

**Supplemental Material 2. Social distancing adherence scale**

Please indicate if you are following the recommended guidelines to protect yourself and others:

**Since the COVID-19 pandemic:**

1. Do you stay at home unless you have to go to work?

1 – Never

2 – Rarely

3 – Sometimes

4 – Very often

5 – Always

1. Do you avoid all non-essential trips in your community?

1 – Never

2 – Rarely

3 – Sometimes

4 – Very often

5 – Always

1. If you leave your home, do you keep a distance of at least 2 arm’s length (approximately 2 metres or 6 feet) from others?

1 – Never

2 – Rarely

3 – Sometimes

4 – Very often

5 – Always

1. Do you limit close contact with people at high risk, such as older adults and those in poor health?

1 – Never

2 – Rarely

3 – Sometimes

4 – Very often

5 – Always

1. Do you avoid crowded places and non-essential gatherings with persons outside of your household (people you live with)?

1 – Never

2 – Rarely

3 – Sometimes

4 – Very often

5 – Always

1. Do you avoid common greetings, such as handshakes (or hugging and kissing)?

1 – Never

2 – Rarely

3 – Sometimes

4 – Very often

5 – Always

**Supplemental Material 3. List of measures**

Primary Outcome Measure

- **Social Distancing Adherence Scale**: a self-report scale developed based on the recommendations from the WHO, CDC, and Public Health Agency of Canada (2–4). The scale consists of 6 items assessed using a Likert scale, ranging from ‘1, Never’ to ‘5, Always’.

***Other Measures***

***Sociodemographic and clinical information***

- Demographic variables (e.g., age, gender, years of education, race, religion, population density, political affiliation, dwelling, employment status, healthcare worker status, household income, marital status, and total number of persons in a household)
- Frequency of substance use (i.e., alcohol, cigarettes, electronic cigarettes, and marijuana/cannabis)

***COVID-19 and social distancing related measures***

- Degree of social support
  - Q. How satisfied are you with your personal relationships? (1, very dissatisfied to 9, very satisfied)
  - Q. How satisfied are you with the support you get from your friends? (1, very dissatisfied to 9, very satisfied)
- Single-item questions to assess the perceived seriousness of COVID-19
  - Q. COVID-19 is serious (1, strongly disagree *to* 5, strongly agree)
- Single-item questions to assess if they know anyone affected by COVID-19 or are at higher risk for COVID-19
  - Q. Do you have someone personally close to you, such as a family member or a friend, who has had COVID-19? What is/was their outcome? (No, I don’t know anyone affected by COVID-19; Mild; Moderate-to-severe without hospitalization; Moderate-to-severe with hospitalization; Required admission to an intensive care unit; Deceased)
  - Q. Do you have someone personally close to you, such as a family member or a friend, who is a health care worker? (no/yes)
  - Q. Do you have someone personally close to you, such as a family member or a friend, who is elderly (greater than 60 years) or has an underlying health condition putting them at higher risk for a negative health outcome due to COVID-19? (no/yes)
  - Q. Do you have someone personally close to you, such as a family member or a friend, who lives in a senior’s residence? (no/yes)
  - Q. Do you have someone personally close to you, such as a family member or a friend, who lives in a long-term care (i.e., nursing) home? (no/yes)
- Single-item questions to assess whether they were tested for COVID-19
  - Q. Have you been tested (e.g., nasopharyngeal, nasal or throat swab) for COVID-19? (no/yes)
  - If yes to above, Q. Was COVID-19 detectable? (no/yes/results pending)
- Single-item question to assess health risk factors for COVID-19
  - Q. Do you have the following conditions: Heart disease (no/yes), hypertension (no/yes), lung disease (no/yes), diabetes (no/yes), cancer (no/yes), chronic kidney disease (no/yes), obesity (no/yes), weakened immune system from medical condition or treatment, such as chemotherapy (no/yes)
- Single-item questions to assess perceptions regarding infection and testing
  - Q. I believe I am infected with COVID-19 (0, strongly disagree *to* 10, strongly agree)
  - Q. I believe I need testing for COVID-19 (0, strongly disagree *to* 10, strongly agree)
- Single-item questions to assess financial impact due to COVID-19
  - Q. My income is reduced due to COVID-19 (1, strongly disagree *to* 5, strongly agree)
- Single-item questions to assess impact of COVID-19 and social distancing measures on mental health
  - Q. Has social distancing affected your mental health? (1, definitely not to 6, definitely)
  - Q. Has COVID-19 affected your mental health? (1, definitely not to 6, definitely)
- Single-item question for source of health information
  - Q. What is your main source of health information? (Friends or family/ Doctor/ Social media/ Internet/ Radio or podcast/Newspaper/ Magazines/ Television/ Other)
- Single-item question to assess belief about the origin of COVID-19
  - Q. What do you believe is the origin of COVID-19? (It came about naturally likely from animals to humans/ It was developed intentionally in a lab/ It was made accidentally in a lab/ It doesn’t really exist/ Other)
- Citizen Trust in Government Organizations Scale (CTGO) (5): A 9-item measure to assess trust in government organizations. Items were tailored to assess trust in government’s management of the COVID-19 pandemic

***Psychological Measures***

- **Risk Propensity Scale (RPS)** (6): A 7-item measure to assess individual’s general tendency to take risks.
- **Perceived Vulnerability to Disease (PVD)** (7): A 15-item measure to assess beliefs about personal susceptibility to contracting an infectious disease.
- **Multidimensional Iowa Suggestibility Scale, Short (MISS)** (8): A 21-item measure to assess suggestibility.
- **Duke Religion/Spirituality Index (DRI), religiosity/spirituality subscale** (9): A measure of religiosity/spirituality. Only the 3-item Intrinsic Religiosity Subscale will be asked for the purpose of this study.
- **Ten-Item Personality Inventory (TIPI)** (10): A brief 10-item measure personality traits, including extraversion, agreeableness, conscientiousness, openness to experience, and emotional stability.
- **Vaccine Attitude Examination (VAX)** (11): A 12-item measure to assess general vaccination attitudes.
- **Holistic Complementary and Alternative Medicine Questionnaire (HCAM)** (12): A 12-item measure that includes six items to assess beliefs about the scientific validity of complementary and alternative medicine and five items to assess to beliefs about holistic health.
- **Brief Locus-of-Control Scale (LOC)** (13): A brief 9-item measure to asses generalized expectancies for internal and external control (chance and powerful others).
- **General Trust scale (GTS)** (14): A 6-item measure to assess an individual’s general trust towards others.
- **Authority Behavior Inventory (ABI)** (1): A 24-item measure to assess an individual’s attitudes or behavior towards authority.
- **Positive and Negative Symptom Scale (PANAS)** (15): A 10-item measure to assess positive and negative feelings and emotions.
- **Experiences in Close Relationships Scale (ECR)** (16): A 16-item measure to assess attachment anxiety and avoidance.

***Scale internal reliability***

| **List of Scales** | **Cronbach’s alpha** |
| --- | --- |
| RPS | 0.71 |
| CTGO | 0.96 |
| PVD |  |
| Germ Aversion | 0.64 |
| Perceived infection | 0.72 |
| MISS | 0.96 |
| DRI | 0.94 |
| TIPI |  |
| Extraversion | 0.45 |
| Agreeableness | 0.27 |
| Conscientiousness | 0.47 |
| Emotional stability | 0.50 |
| Openness | 0.20 |
| VAX | 0.86 |
| HCAM | 0.42 |
| LOC |  |
| Internal | 0.77 |
| Chance | 0.82 |
| Powerful others | 0.90 |
| GTS | 0.90 |
| ABI | 0.63 |
| PANAS |  |
| Positive symptoms | 0.92 |
| Negative symptoms | 0.94 |
| ECR |  |
| Attachment anxiety | 0.91 |
| Avoidance | 0.71 |

**Supplemental Material 4.** Sociodemographic, COVID-19 and social distancing related, and psychological characteristics in men, women, and participants 60 years of age or older.

|  | **Mean (SD), range or N (%)** | | | |
| --- | --- | --- | --- | --- |
|  | **All** | **Men** | **Women** | $\boldsymbol{\geq}$**60** |
| **N** | **4942** | **2419** | **2499** | **1496** |
| Social Distancing Adherence score | 4.3 (0.7), 1.0-5.0 | 4.2 (0.8), 1.0-5.0 | 4.4 (0.7), 1.0-5.0 | 4.5 (0.6), 1.0-5.0 |
| **Sociodemographic determinants** |  |  |  |  |
| Age | 44.7 (17.3) | 49.12 (17.0) | 46.5 (17.5) | 68.8 (6.1) |
| Gender (man/woman) | 2419 (49.2)/2499 (50.8) | N/A | N/A | 772 (51.7)/721 (48.3) |
| Education (years) | 15.2 (3.9) | 15.4 (4.1) | 15.1 (3.7) | 15.6 (3.3) |
| Race |  |  |  |  |
| Indigenous (First Nations, Inuit or Métis) | 48 (1.0) | 24 (1.0) | 23 (0.9) | 11 (0.7) |
| Black | 219 (4.4) | 90 (3.7) | 128 (5.1) | 41 (2.7) |
| East Asian | 529 (10.7) | 240 (9.9) | 287 (11.5) | 60 (4.0) |
| Latinx | 338 (6.8) | 147 (6.1) | 190 (7.6) | 57 (3.8) |
| South Asian | 132 (2.7) | 72 (3.0) | 60 (2.4) | 13 (0.9) |
| Other | 376 (7.6) | 165 (6.8) | 205 (8.2) | 58 (3.9) |
| White | 3300 (66.8) | 1681 (69.5) | 1606 (64.3) | 1256 (84.0) |
| Religion (yes/no) | 3133 (66.2)/1602 (33.8) | 1560 (64.5)/771 (31.9) | 1564 (65.7)/818 (34.3) | 1079 (74.6)/367 (25.4) |
| Population density |  |  |  |  |
| 1,000 or less | 128 (2.9) | 61 (2.5) | 67 (3.1) | 42 (3.2) |
| 1,000 to 29,999 | 491 (11.1) | 229 (10.2) | 258 (12.1) | 137 (10.3) |
| 30,000 to 99,999 | 768 (17.4) | 386 (17.1) | 379 (17.1) | 218 (16.4) |
| 100,000 or more | 3025 (68.6) | 1578 (70.0) | 1436 (67.1) | 933 (70.2) |
| Political spectrum |  |  |  |  |
| Communism left wing or socialism | 281 (5.7) | 138 (5.7) | 131 (5.2) | 55 (3.7) |
| Liberal | 1452 (29.4) | 662 (27.4) | 784 (31.4) | 416 (27.8) |
| Center | 1758 (35.6) | 790 (32.7) | 963 (38.5) | 483 (32.3) |
| Conservative | 1356 (27.4) | 764 (31.6) | 591 (23.6) | 538 (36.0) |
| Fascism right wing or authoritarianism | 95 (1.9) | 65 (2.7) | 30 (1.2) | 4 (0.3) |
| Employment status |  |  |  |  |
| Unemployed | 595 (12.0) | 211 (8.7) | 379 (15.2) | 69 (4.6) |
| Employed | 2735 (55.3) | 1467 (60.6) | 1260 (50.4) | 363 (24.3) |
| Student | 281 (5.7) | 93 (3.8) | 184 (7.4) | 1 (0.1) |
| Retired | 1093 (22.1) | 572 (23.6) | 519 (20.8) | 998 (66.7) |
| Other | 238 (4.8) | 76 (3.1) | 157 (6.3) | 65 (4.3) |
| Healthcare worker status (yes/no) | 669 (13.9)/4273 (86.5) | 326 (13.5)/2093 (86.5) | 341 (13.6)/2158 (86.4) | 125 (8.4)/1371 (91.6) |
| Dwelling |  |  |  |  |
| House with a backyard | 3043 (61.6) | 1526 (63.1) | 1506 (60.3) | 926 (61.9) |
| House without a backyard | 156 (3.2) | 89 (3.7) | 67 (2.7) | 25 (1.7) |
| Apartment‎/condominium‎/loft with no or small private outdoor space | 1275 (25.8) | 570 (23.6) | 695 (27.8) | 391 (26.1) |
| Apartment‎/condominium‎/loft with large outdoor space | 352 (7.1) | 179 (7.4) | 173 (6.9) | 122 (8.2) |
| Senior's residence | 24 (0.5) | 12 (0.5) | 11 (0.4) | 13 (0.9) |
| Long-term facility‎ or‎ nursing home | 5 (0.1) | 3 (0.1) | 2 (0.1) | 1 (0.1) |
| Other or not reported | 87 (1.8) | 40 (1.7) | 45 (1.8) | 18 (1.2) |
| Household income |  |  |  |  |
| Less than $20,000 | 319 (6.9) | 129 (5.6) | 187 (8.1) | 82 (5.9) |
| $20,000 - $59,999 | 1225 (26.4) | 497 (21.5) | 720 (31.2) | 405 (29.1) |
| $60,000 - $99,999 | 1364 (29.4) | 683 (29.5) | 677 (29.3) | 420 (30.2) |
| $100,000 - $139,999 | 815 (17.6) | 445 (19.2) | 370 (16.0) | 221 (15.9) |
| $140,000 or more | 918 (19.8) | 560 (24.2) | 356 (15.4) | 265 (19.0) |
| Marital status (single/married) | 2223 (45.0)/719 (55.0) | 944 (39.0)/1475 (61.1) | 1261 (50.5)/1238 (49.5) | 547 (36.6)/949 (63.4) |
| Number of persons in a household | 2.5 (1.5), 1.0-55.0 | 2.5 (1.3), 1.0-21.0 | 2.5 (1.3), 1.0-10.0 | 2.0 (0.9), 1.0-7.0 |
| Substance use per week |  |  |  |  |
| Alcohol use (yes/no) | 3146 (63.7)/1796 (36.3) | 1637 (67.7)/782 (32.3) | 1501 (60.1)/998 (39.9) | 956 (63.9)/540 (36.1) |
| Cigarette use (yes/no) | 929 (18.8)/4013 (81.2) | 551 (33.8)/1868 (77.2) | 377 (15.1)/2122 (84.9) | 170 (11.4)/1326 (88.6) |
| E-cigarette use (yes/no) | 635 (12.8)/4307 (87.2) | 407 (16.8)/2012 (83.2) | 224 (9.0)/2275 (91.0) | 38 (2.5)/1458 (97.5) |
| Cannabis use (yes/no) | 873 (17.7)/4069 (82.3) | 495 (20.5)/1924 (79.5) | 373 (14.9)/2126 (85.1) | 162 (10.8)/1334 (89.2) |
| **COVID-19 and social distancing related determinants** |  |  |  |  |
| Degree of social support (total score^1^) | 13.7 (3.7), 2.0-18.0 | 13.8 (3.7), 2.0-18.0 | 13.6 (3.8), 2.0-18.0 | 14.7 (3.4), 2.0-18.0 |
| Perceived seriousness of COVID-19 | 4.4 (0.9), 1.0-5.0 | 4.4 (1.0), 1.0-5.0 | 4.5 (0.9), 1.0-5.0 | 4.4 (0.7), 1.0-5.0 |
| Knowing someone personally close who |  |  |  |  |
| …is a healthcare worker (yes/no) | 1852 (37.5)/3090 (62.5) | 860 (35.6)/1559 (64.4) | 986 (39.5)/1513 (60.5) | 471 (31.5)/1025 (68.5) |
| …is elderly (>60 years) or has underlying health condition (yes/no) | 3131 (63.4)/1811 (36.6) | 1451 (60.0)/968 (40.0) | 1664 (66.6)/835 (33.4) | 1086 (72.6)/410 (27.4) |
| …lives in a senior’s residence (yes/no) | 1016 (20.6)/3926 (79.4) | 514 (21.2)/1905 (78.8) | 497 (19.9)/2002 (80.1) | 271 (18.1)/1225 (81.9) |
| …lives in a long-term care home (yes/no) | 862 (17.4)/4080 (82.6) | 445 (18.4)/1974 (81.6) | 415 (16.6)/2084 (83.4) | 197 (13.2)/1299 (86.8) |
| …has had COVID-19 and their outcome |  |  |  |  |
| With mild symptoms | 467 (9.4) | 219 (9.1) | 246 (9.8) | 113 (7.6) |
| Moderate-to-severe without hospitalization | 427 (8.6) | 206 (8.5) | 217 (8.7) | 101 (6.8) |
| Moderate-to-severe with hospitalization | 211 (4.3) | 112 (4.6) | 98 (3.9) | 42 (2.8) |
| Required admission to an intensive care unit | 107 (2.2) | 51 (2.1) | 56 (2.2) | 15 (1.0) |
| Deceased | 189 (3.8) | 87 (3.6) | 102 (4.1) | 64 (4.3) |
| Does not know anyone affected | 3541 (71.7) | 1744 (72.1) | 1780 (71.2) | 1161 (77.6) |
| Prior laboratory testing for COVID-19 |  |  |  |  |
| Tested + | 128 (2.6) | 85 (3.5) | 43 (1.7) | 4 (0.3) |
| Tested - | 590 (11.9) | 321 (13.3) | 267 (10.7) | 152 (10.2) |
| Tested and pending | 37 (0.7) | 25 (1.0) | 11 (0.4) | 12 (0.8) |
| Never tested | 4187 (97.4) | 1988 (82.2) | 2178 (87.2) | 1328 (88.8) |
| COVID-19 health risk factors (total score^2^) | 0.7 (1.1), 0.0-8.0 | 0.8 (1.2), 0.0-8.0 | 0.6 (1.0), 0.0-8.0 | 1.1 (1.1), 0.0-7.0 |
| Believing one is infected with COVID-19 | 1.0 (2.3), 0.0-10.0 | 1.2 (2.6), 0.0-10.0 | 0.8 (2.0), 0.0-10.0 | 0.4 (1.4), 0.0-10.0 |
| Believing one need testing for COVID-19 | 2.5 (3.2), 0.0-10.0 | 2.9 (2.2), 0.0-10.0 | 2.1 (2.9), 0.0-10.0 | 1.9 (2.7), 0.0-10.0 |
| Reduction in income due to COVID-19 | 2.7 (1.5), 1.0-5.0 | 2.7 (1.5), 1.0-5.0 | 2.7 (1.4), 1.0-5.0 | 2.2 (1.3), 1.0-5.0 |
| Negative impact of social distancing on mental health | 2.9 (1.7), 1.0-6.0 | 2.8 (1.6), 1.0-6.0 | 3.1 (1.7), 1.0-6.0 | 2.2 (1.4), 1.0-6.0 |
| Negative impact of COVID-19 on mental health | 2.8 (1.6), 1.0-6.0 | 2.7 (1.6), 1.0-6.0 | 3.0 (1.7), 1.0-6.0 | 2.2 (1.4), 1.0-6.0 |
| Source of health information |  |  |  |  |
| Friends or family | 247 (5.0) | 106 (4.4) | 138 (5.5) | 29 (1.9) |
| Doctor | 1210 (24.5) | 663 (27.4) | 542 (21.7) | 417 (27.9) |
| Social media | 331 (6.7) | 150 (6.2) | 179 (7.2) | 16 (1.1) |
| Internet | 1403 (28.4) | 677 (28.0) | 716 (28.7) | 251 (16.8) |
| Radio/Podcast | 150 (3.0) | 82 (3.4) | 67 (2.7) | 34 (2.3) |
| Newspaper | 263 (5.3) | 132 (5.5) | 130 (5.2) | 115 (7.7) |
| Magazines | 17 (0.3) | 7 (0.3) | 10 (0.4) | 2 (0.1) |
| Television | 1216 (24.6) | 560 (23.2) | 655 (26.2) | 594 (39.7) |
| Other | 105 (2.1) | 42 (1.7) | 62 (2.5) | 38 (2.5) |
| Origin of COVID-19 |  |  |  |  |
| It was developed intentionally in a lab | 951 (19.2) | 492 (20.3) | 457 (18.3) | 230 (15.4) |
| It was made accidentally in a lab | 413 (8.4) | 215 (8.9) | 198 (7.9) | 145 (9.7) |
| It doesn't really exist | 58 (1.2) | 34 (1.4) | 24 (1.0) | 9 (0.6) |
| It came about naturally likely from animals to humans | 3191 (64.6) | 1546 (63.9) | 1624 (65.0) | 973 (65.0) |
| Other/I don’t know | 329 (6.7) | 132 (5.5) | 196 (7.8) | 139 (9.3) |
| CTGO, trust in government’s management of COVID-19 | 22.3 (8.9), 8.0-40.0 | 22.8 (9.0), 8.0-40.0 | 21.8 (8.8), 8.0-40.0 | 20.4 (8.9), 8.0-40.0 |
| **Psychological determinants** |  |  |  |  |
| RPS, Risk propensity | 24.1 (8.7), 7.0-61.0 | 25.8 (8.5), 7.0-61.0 | 22.5 (8.7), 7.0-51.0 | 21.3 (8.4), 7.0-49.0 |
| PVD, Germ aversion subscale | 4.8 (1.0), 1.4-7.0 | 4.6 (0.9), 1.5-7.0 | 4.9 (1.0), 1.4-7.0 | 4.9 (1.1), 1.5-7.0 |
| PVD, Perceived infectability subscale | 3.5 (1.1), 1.0-7.0 | 3.4 (1.0), 1.0-7.0 | 3.5 (1.2), 1.0-7.0 | 3.1 (1.1), 1.0-7.0 |
| MISS, Suggestibility | 45.4 (18.0), 21.0-105.0 | 46.3 (19,4), 21.0-105.0 | 44.5 (16.5), 21.0-105.0 | 35.8 (10.0), 21.0-105.0 |
| DRI, Religiosity/spirituality subscale | 8.6 (4.1), 3.0-15.0 | 8.6 (4.1), 3.0-15.0 | 8.7 (4.0), 3.0-15.0 | 8.7 (4.1), 3.0-15.0 |
| TIPI, Extraversion | 3.8 (1.4), 1.0-7.0 | 3.7 (1.3), 1.0-7.0 | 3.8 (1.4), 1.0-7.0 | 3.9 (1.5), 1.0-7.0 |
| TIPI, Agreeableness | 4.9 (1.2), 1.0-7.0 | 4.7 (1.1), 1.0-7.0 | 5.0 (1.2), 1.0-7.0 | 5.3 (1.1), 1.5-7.0 |
| TIPI, Conscientiousness | 5.3 (1.3), 1.0-7.0 | 5.3 (1.3), 1.0-7.0 | 5.4 (1.3), 1.0-7.0 | 5.9 (1.1), 1.5-7.0 |
| TIPI, Emotional stability | 4.7 (1.3), 1.0-7.0 | 4.8 (1.2), 1.0-7.0 | 4.6 (1.4), 1.0-7.0 | 5.2 (1.3), 1.0-7.0 |
| TIPI, Openness to experience | 4.6 (1.1), 1.0-7.0 | 4.6 (1.1), 1.0-7.0 | 4.7 (1.2), 1.0-7.0 | 4.7 (1.2), 1.0-7.0 |
| VAX total score^3^ | 3.1 (1.0), 1.0-6.0 | 3.1 (1.0), 1.0-6.0 | 3.1 (1.0), 1.0-6.0 | 2.9 (1.0), 1.0-6.0 |
| HCAM, Holistic health subscale^4^ | 12.1 (4.4), 5.0-30.0 | 12.5 (4.5), 5.0-30.0 | 11.7 (4.2), 5.0-30.0 | 11.1 (3.6), 5.0-30.0 |
| HCAM, Complementary and alternative medicine subscale^4^ | 23.4 (4.7), 6.0-36.0 | 23.6 (4.5), 6.0-36.0 | 23.2 (4.8), 6.0-36.0 | 24.3 (5.2), 6.0-36.0 |
| LOC, Internal | 15.4 (3.3), 3.0-21.0 | 15.6 (3.3), 3.0-21.0 | 15.2 (3.2), 3.0-21.0 | 15.9 (3.0), 3.0-21.0 |
| LOC, Chance | 11.4 (4.1), 3.0-21.0 | 11.6 (4.3), 3.0-21.0 | 11.1 (3.9), 3.0-21.0 | 9.7 (3.6), 3.0-21.0 |
| LOC, Powerful others | 10.4 (4.8), 3.0-21.0 | 10.8 (4.9), 3.0-21.0 | 9.9 (4.7), 3.0-21.0 | 8.3 (4.2), 3.0-21.0 |
| GTS, General trust | 3.5 (0.8), 1.0-5.0 | 3.6 (0.7), 1.0-5.0 | 3.4 (0.8), 1.0-5.0 | 3.7 (0.7), 1.0-5.0 |
| ABI, Behavior towards authority | 77.8 (8.2), 42.0-108.0 | 77.1 (7.8), 45.0-107.0 | 78.6 (8.5), 42.0-108.0 | 81.3 (8.2), 48.0-108.0 |
| PANAS, Positive affect score | 32.3 (8.1), 10.0-50.0 | 33.0 (8.1), 10.0-50.0 | 31.7 (8.1), 10.0-50.0 | 33.6 (7.1), 10.0-50.0 |
| PANAS, Negative affect score | 20.4 (8.9), 10.0-50.0 | 20.5 (9,3), 10.0-50.0 | 20.2 (8.4), 10.0-50.0 | 16.3 (5.9), 10.0-50.0 |
| ECR, Attachment anxiety | 28.9 (11.1), 8.0-56.0 | 29.3 (11.1), 8.0-56.0 | 28.6 (11.2), 8.0-56.0 | 23.6 (9.3), 8.0-56.0 |
| ECR, Attachment avoidance | 29.7 (7.5), 8.0-56.0 | 30.2 (6.9), 8.0-56.0 | 29.1 (8.0), 8.0-56.0 | 28.4 (8.2), 8.0-56.0 |

CTGO, Citizen Trust in Government Organizations’ Scale; RPS, Risk Propensity Scale; PVD, Perceived Vulnerability to Disease Questionnaire; MISS, Multidimensional Iowa Suggestibility Scale; DRI, Duke Religion/Spirituality Index; TIPI, Ten-Item Personality Inventory; VAX, Vaccination Attitudes Examination Scale; HCAM, Holistic Complementary and Alternative Medicine Questionnaire; LOC, Brief Locus-of-Control Scale; GTS, General Trust Scale; ABI, Authority Behavior Index; PANAS, Positive and Negative Affect Schedule; ECR, Experiences in Close Relationships Scale.

^1^A total score was derived from adding scores for the degree of satisfaction with personal relationships and support from friends.

^2^One point was assigned for each health risk factor (i.e., heart disease, hypertension, lung disease, diabetes, cancer, chronic kidney disease, obesity, and weakened immune system) to derive a total health risk factor score for COVID-19.

^3^Higher scores represent anti-vaccination attitudes.

^4^Higher scores represent a more negative attitude toward holistic complementary and alternative medicine.

**Supplemental Material 5.** Associations between sociodemographic, COVID-19 and social distancing related, and psychological determinants and social distancing adherence score in men, women, and participants 60 years of age or older.

|  | **r (df) or F, p-value** | | | |
| --- | --- | --- | --- | --- |
|  | **All** | **Men** | **Women** | $\boldsymbol{\geq}$**60** |
| **N** | **4942** | **2419** | **2499** | **1496** |
| **Sociodemographic determinants** |  |  |  |  |
| Age | r(4942)=0.20, p<0.001 | r(2419)=0.19, p<0.001 | r(2499)=0.23, p<0.001 | r(1496)=0.90, p=0.001 |
| Gender (man/woman) | F(1,4916)=44.78, p<0.001 | N/A | N/A | F(1,1491)=39.96,p<0.001 |
| Education (years) | r(4939)=0.55, p<0.001 | r(2418)=0.08, p<0.001 | r(2497)=0.04, p=0.065 | r(1494)=-0.00, p=0.893 |
| Race | F(6,4941)=3.05, p=0.006 | F(6,2418)=1.29, p=0.257 | F(6,2498)=3.17, p=0.004 | F(6,1495)=0.90, p=0.495 |
| Religion (yes/no) | F(1,4733)=2.0, p=0.157 | F(1,2329)=0.00, p=0.993 | F(1,2380)=4.97, p=0.026 | F(1,1444)=0.54, p=0.462 |
| Population density | r(4412)=0.06, p<0.001 | r(2254)=0.06, p=0.005 | r(2140)=0.06, p=0.006 | r(1330)=0.00, p=0.985 |
| Political spectrum | F(4,4937)=22.42, p<0.001 | F(4,2414)=10.90, p<0.001 | F(2,2494)=10.46,p<0.001 | F(4,1491)=13.72,p=0.001 |
| Employment status | F(4,4937)=30.70, p<0.001 | F(4,2414)=17.73, p<0.001 | F(4,2494)=22.09,p<0.001 | F(4,1491)=1.12, p=0.346 |
| Healthcare worker status (yes/no) | F(1,4941)=13.74, p<0.001 | F(1,2418)=11.09, p=0.001 | F(1,2498)=3.72, p=0.054 | F(1,1495)=0.24, p=0.624 |
| Dwelling | F(6,4892)=1.49, p=0.178 | F(6,2389)=0.96, p=0.452 | F(6,2472)=1.46, p=0.188 | F(6,1486)=0.50, p=0.811 |
| Household income | r(4641)=-0.00, p=0.785 | r(2314)=0.04, p=0.058 | r(2310)=-0.02, p=0.307 | r(1393)=-0.08, p=0.004 |
| Marital status (single/married) | F(1, 4940)=7.12, p=0.008 | F(1,2417)=6.03, p=0.014 | F(1,2497)=6.56, p=0.010 | F(1,1494)=0.11, p=0.746 |
| Number of persons in a household | r(4880)=-0.03, p=0.043 | r(2391)=-0.05, p=0.024 | r(2467)=-0.00, p=0.897 | r(1490)=-0.00, p=0.875 |
| Substance use per week |  |  |  |  |
| Alcohol use (yes/no) | F(1,4940)=4.57, p=0.033 | F(1,2417)=0.56, p=0.455 | F(1, 2497)=1.90, p=0.168 | F(1,1494)=5.30, p=0.021 |
| Cigarette use (yes/no) | F(1,4940)=49.05, p<0.001 | F(1,2417)=40.86, p<0.001 | F(1,2497)=4.82, p=0.028 | F(1,1494)=2.42, p=0.120 |
| E-cigarette use (yes/no) | F(1,4940)=97.85, p<0.001 | F(1,2417)=42.86, p<0.001 | F(1,2497)=40.02,p<0.001 | F(1,1494)=4.91, p=0.027 |
| Cannabis use (yes/no) | F(1,4940)=14.68, p<0.001 | F(1,2417)=3.00, p=0.084 | F(1,2497)=8.84, p=0.003 | F(1,1492)=0.05, p=0.833 |
| **COVID-19 and social distancing related determinants** |  |  |  |  |
| Degree of social support (total score^1^) | r(4838)=0.22, p<0.001 | r(2367)=0.22, p<0.001 | r(2447)=0.22, p<0.001 | r(1450)=-0.15, p<0.001 |
| Perceived seriousness of COVID-19 | r(3923)=0.48, p<0.001 | r(1913)=0.49, p<0.001 | r(1991)=0.45, p<0.001 | r(1210)=0.36, p<0.001 |
| Knowing someone personally close who |  |  |  |  |
| …is a healthcare worker (yes/no) | F(1,4940)=0.39, p=0.532 | F(1,2417)=0.17, p=0.681 | F(1,2497)=0.00, p=0.951 | F(1,1494)=0.01, p=0.767 |
| …is elderly (>60 years) or has underlying health condition (yes/no) | F(1,4940)=93.65, p<0.001 | F(1,2417)=57.82, p<0.001 | F(1,2497)=29.78,p<0.001 | F(1,1494)=13.85,p<0.001 |
| …lives in a senior’s residence (yes/no) | F(1,4940)=7.03, p=0.008 | F(1,2417)=0.32, p=0.570 | F(1,2497)=9.60, p=0.002 | F(1,1494)=1.15, p=0.284 |
| …lives in a long-term care home (yes/no) | F(1,4940)=17.81, p<0.001 | F(1,2417)=3.19, p=0.074 | F(1,2497)=16.47,p<0.001 | F(1,1494)=1.00, p=0.317 |
| …has had COVID-19 and their outcome | F(5,4936)=8.18, p<0.001 | F(5,2413)=5.93, p<0.001 | F(5,2493)=3.27, p=0.006 | F(5,1490)=1.33, p=0.251 |
| Prior laboratory testing for COVID-19 | F(3,2938)=12.06, p<0.001 | F(3,2415)=2.68, p=0.05 | F(3,2495)=11.66,p<0.001 | F(3,1492)=0.22, p=0.882 |
| COVID-19 health risk factors (total score^2^) | r(4942)=0.06, p<0.001 | r(2419)=0.09, p<0.001 | r(2499)=0.05, p=0.008 | r(1496)=0.06, p=0.028 |
| Believing one is infected with COVID-19 | r(4942)=-0.21, p<0.001 | r(2419)=-0.21, p<0.001 | r(2499)=-0.19, p<0.001 | r(1496)=-0.09, p=0.001 |
| Believing one need testing for COVID-19 | r(4942)=0.41, p<0.001 | r(2419)=-0.12, p<0.001 | r(2499)=-0.15, p<0.001 | r(1496)=-0.09, p=0.001 |
| Reduction in income due to COVID-19 | r(4942)=-0.05, p=0.001 | r(2419)=-0.05, p=0.009 | r(2499)=-0.03, p=0.126 | r(1496)=-0.04, p=0.147 |
| Negative impact of social distancing on mental health | r(4838)=-0.13, p<0.001 | r(2367)=-0.15, p<0.001 | r(2477)=-0.14, p<0.001 | r(1450)=-0.07, p=0.005 |
| Negative impact of COVID-19 on mental health | r(4838)=-0.08, p<0.001 | r(2367)=-0.09, p<0.001 | r(2477)=-0.09, p<0.001 | r(1450)=-0.03, p=0.266 |
| Source of health information | F(8,4933)=14.92, p<0.001 | F(8,2410)=8.93, p<0.001 | F(8,2490)=7.47, p<0.001 | F(8,1487)=1.66, p=0.104 |
| Origin of COVID-19 | F(4,4937)=62.11, p<0.001 | F(4,2414)=38.05, p<0.001 | F(4,2494)=22.62,p<0.001 | F(4,1491)=13.52,p<0.001 |
| CTGO, trust in government’s management of COVID-19 | r(4942)=0.02, p=0.303 | r(2419)=0.02, p=0.338 | r(2499)=0.03, p=0.164 | r(1496)=0.05, p=0.054 |
| **Psychological determinants** |  |  |  |  |
| RPS, Risk propensity | r(4942)=-0.38, p<0.001 | r(2419)=-0.37, p<0.001 | r(2499)=-0.36, p<0.001 | r(1496)=-0.31, p<0.001 |
| PVD, Germ aversion subscale | r(4942)=0.30, p<0.001 | r(2419)=0.30, p<0.001 | r(2499)=0.28, p<0.001 | r(1496)=0.22, p<0.001 |
| PVD, Perceived infectability subscale | r(4942)=-0.06, p<0.001 | r(2419)=-0.06, p=0.003 | r(2499)=-0.06, p=0.005 | r(1496)=0.05, p=0.075 |
| MISS, Suggestibility | r(4942)=-0.18, p<0.001 | r(2419)=-0.15, p<0.001 | r(2499)=-0.20, p<0.001 | r(1496)=-0.05, p=0.044 |
| DRI, Religiosity/spirituality subscale | r(4942)=0.01, p=0.658 | r(2419)=-0.01, p=0.682 | r(2499)=0.02, p=0.442 | r(1496)=0.04, p=0.159 |
| TIPI, Extraversion | r(4942)=-0.02, p=0.257 | r(2419)=-0.03, p=0.090 | r(2499)=-0.00, p=0.852 | r(1496)=-0.03, p=0.329 |
| TIPI, Agreeableness | r(4942)=0.23, p<0.001 | r(2419)=0.21, p<0.001 | r(2499)=0.23, p<0.001 | r(1496)=0.21, p<0.001 |
| TIPI, Conscientiousness | r(4942)=0.25, p<0.001 | r(2419)=0.25, p<0.001 | r(2499)=0.24, p<0.001 | r(1496)=0.19, p<0.001 |
| TIPI, Emotional stability | r(4942)=0.15, p<0.001 | r(2419)=0.18, p<0.001 | r(2499)=0.15, p<0.001 | r(1496)=0.08, p=0.004 |
| TIPI, Openness to experience | r(4942)=0.16, p<0.001 | r(2419)=0.17, p<0.001 | r(2499)=0.14, p<0.001 | r(1496)=0.08, p=0.001 |
| VAX total score^3^ | r(4942)=-0.15, p<0.001 | r(2419)=-0.17, 0<0.001 | r(2499)=-0.13, p<0.001 | r(1496)= -0.06, p=0.013 |
| HCAM, Holistic health subscale^4^ | r(4942)=-0.22, p<0.001 | r(2419)=-0.21, p<0.001 | r(2499)=-0.22, p<0.001 | r(1496)=-0.11, p<0.001 |
| HCAM, Complementary and alternative medicine subscale^4^ | r(4942)=0.15, p<0.001 | r(2419)=0.19, p<0.001 | r(2499)=0.13, p<0.001 | r(1496)=0.09, p=0.001 |
| LOC, Internal | r(4942)=0.18, p<0.001 | r(2419)=0.19, p<0.001 | r(2499)=0.18, p<0.001 | r(1496)=0.10, p<0.001 |
| LOC, Chance | r(4942)=-0.11, p<0.001 | r(2419)=-0.11, p<0.001 | r(2499)=-0.10, p<0.001 | r(1496)=-0.05, p=0.037 |
| LOC, Powerful others | r(4942)=-0.15, p<0.001 | r(2419)=-0.13, p<0.001 | r(2499)=-0.16, p<0.001 | r(1496)=-0.07, p=0.005 |
| GTS, General trust | r(4942)=0.14, p<0.001 | r(2419)=0.18, p<0.001 | r(2499)=0.12, p<0.001 | r(1496)=0.08, p=0.003 |
| ABI, Behavior towards authority | r(4942)=0.20, p<0.001 | r(2419)=0.19, p<0.001 | r(2499)=0.19, p<0.001 | r(1496)=0.10, p<0.001 |
| PANAS, Positive affect score | r(4942)=0.14, p<0.001 | r(2419)=0.15, p<0.001 | r(2499)=0.15, p<0.001 | r(1496)=0.10, p<0.001 |
| PANAS, Negative affect score | r(4942)=-0.19, p<0.001 | r(2419)=-0.19, p<0.001 | r(2499)=-0.29, p<0.001 | r(1496)=0.06, p=0.029 |
| ECR, Attachment anxiety | r(4942)=-0.15, p<0.001 | r(2419)=-0.12, p<0.001 | r(2499)=-0.17, p<0.001 | r(1496)=-0.12, p<0.001 |
| ECR, Attachment avoidance | r(4942)=-0.05, p<0.001 | r(2419)=-0.03, p=0.138 | r(2499)=-0.05, p=0.015 | r(1496)=-0.01, p=0.628 |

CTGO, Citizen Trust in Government Organizations’ Scale; RPS, Risk Propensity Scale; PVD, Perceived Vulnerability to Disease Questionnaire; MISS, Multidimensional Iowa Suggestibility Scale; DRI, Duke Religion/Spirituality Index; TIPI, Ten-Item Personality Inventory; VAX, Vaccination Attitudes Examination Scale; HCAM, Holistic Complementary and Alternative Medicine Questionnaire; LOC, Brief Locus-of-Control Scale; GTS, General Trust Scale; ABI, Authority Behavior Index; PANAS, Positive and Negative Affect Schedule; ECR, Experiences in Close Relationships Scale.

^1^A total score was derived from adding scores for the degree of satisfaction with personal relationships and support from friends.

^2^One point was assigned for each health risk factor (i.e., heart disease, hypertension, lung disease, diabetes, cancer, chronic kidney disease, obesity, and weakened immune system) to derive a total health risk factor score for COVID-19.

^3^Higher scores represent anti-vaccination attitudes.

^4^Higher scores represent a more negative attitude toward holistic complementary and alternative medicine.

**Supplemental Material 6.** Univariate analysis examining the association between sociodemographic determinants and social distancing adherence in men.^1^

|  | **Beta** | **SE** | **t** | **p-value** | **Partial η^2^** |
| --- | --- | --- | --- | --- | --- |
| Age | 0.01 | 0.00 | 5.44 | <0.001* | 0.01^a^ |
| Race |  |  |  |  |  |
| Indigenous | 0.08 | 0.15 | 0.54 | 0.588 | 0.00 |
| Black | 0.02 | 0.08 | 0.24 | 0.813 | 0.00 |
| East Asian | 0.04 | 0.05 | 0.83 | 0.408 | 0.00 |
| Latinx | 0.18 | 0.07 | 2.69 | 0.007* | 0.00 |
| South Asian | 0.25 | 0.09 | 2.77 | 0.006* | 0.00 |
| Other | 0.10 | 0.06 | 1.60 | 0.111 | 0.00 |
| White^2^ | - | - | - | - | - |
| Education (years) | 0.01 | 0.00 | 1.41 | 0.159 | 0.00 |
| Region of residence |  |  |  |  |  |
| Canada | 0.10 | 0.04 | 2.89 | 0.004* | 0.00 |
| Florida/Texas | -0.01 | 0.04 | -0.34 | 0.734 | 0.00 |
| New York/California^2^ | - | - | - | - | - |
| Religion (yes/no^2^) | 0.01 | 0.03 | 0.40 | 0.691 | 0.00 |
| Population density |  |  |  |  |  |
| 1,000 or less | -0.09 | 0.10 | -0.96 | 0.338 | 0.00 |
| 1,000 to 29,999 | -0.10 | 0.05 | -1.82 | 0.068 | 0.00 |
| 30,000 to 99,999 | 0.01 | 0.04 | 0.24 | 0.810 | 0.00 |
| 100,000 or more^2^ | - | - | - | - | - |
| Political affiliation |  |  |  |  |  |
| Communism left wing or socialism | 0.18 | 0.07 | 2.64 | 0.008* | 0.00 |
| Liberal | 0.17 | 0.04 | 4.33 | <0.001* | 0.01^a^ |
| Center^2^ | - | - | - | - | - |
| Conservative | -0.08 | 0.04 | -2.20 | 0.028 | 0.00 |
| Fascism right wing or authoritarianism | 0.08 | 0.10 | 0.77 | 0.441 | 0.00 |
| Healthcare worker status (yes/no^2^) | -0.05 | 0.05 | -1.02 | 0.306 | 0.00 |
| Employment status |  |  |  |  |  |
| Unemployed | 0.16 | 0.06 | 2.70 | 0.007* | 0.00 |
| Employed^2^ | - | - | - | - | - |
| Student | 0.12 | 0.09 | 1.38 | 0.167 | 0.00 |
| Retired | 0.09 | 0.05 | 1.81 | 0.070 | 0.00 |
| Dwelling |  |  |  |  |  |
| House with a backyard^2^ | - | - | - | - | - |
| House without a backyard | 0.03 | 0.08 | 0.36 | 0.721 | 0.00 |
| Apartment‎/condominium‎/loft with no or small private outdoor space | -0.06 | 0.04 | -1.66 | 0.098 | 0.00 |
| Apartment‎/condominium‎/loft with large outdoor space | 0.02 | 0.06 | 0.36 | 0.720 | 0.00 |
| Senior's residence | 0.23 | 0.21 | 1.10 | 0.270 | 0.00 |
| Long-term facility‎ or‎ nursing home | 0.82 | 0.42 | 1.94 | 0.053 | 0.00 |
| Household income |  |  |  |  |  |
| Less than $20,000 | -0.13 | 0.08 | -1.73 | 0.084 | 0.00 |
| $20,000-$59,999 | -0.07 | 0.05 | -1.52 | 0.129 | 0.00 |
| $60,000-$99,999^2^ | - | - | - | - | - |
| $100,000-$139,999 | 0.01 | 0.05 | 0.20 | 0.846 | 0.00 |
| $140,000 or more | 0.06 | 0.04 | 1.27 | 0.205 | 0.00 |
| Marital status (single/married^2^) | 0.00 | 0.04 | -0.02 | 0.986 | 0.00 |
| Number of persons in a household | 0.00 | 0.01 | 0.09 | 0.931 | 0.00 |
| Substance use in the past week |  |  |  |  |  |
| Alcohol use (yes/no^2^) | -0.02 | 0.03 | -0.51 | 0.610 | 0.00 |
| Cigarette use (yes/no^2^) | -0.13 | 0.05 | -2.58 | 0.010 | 0.00 |
| Electronic cigarette use (yes/no^2^) | -0.08 | 0.06 | -1.41 | 0.159 | 0.00 |
| Cannabis use (yes/no^2^) | 0.04 | 0.04 | 1.01 | 0.311 | 0.00 |

^1^Total adjusted R^2^: 0.08; ^2^Reference variable.

^a^Small effect (η^2^=0.01); ^b^Medium effect (η^2^=0.06); ^c^Large effect (η^2^=0.14); *p<0.01 (0.05/3 univariate models)

**Supplemental Material 7.** Univariate analysis examining the association between COVID-19 and social distancing determinants and social distancing adherence in men.^1^

|  | **Beta** | **SE** | **t** | **p-value** | **Partial η^2^** |
| --- | --- | --- | --- | --- | --- |
| Degree of social support (total score^3^) | 0.03 | 0.00 | 6.18 | 0.000 | 0.02^a^ |
| Perceived seriousness of COVID-19 | 0.38 | 0.02 | 22.16 | 0.000 | 0.21^c^ |
| Knowing someone personally close who |  |  |  |  |  |
| …is a healthcare worker (yes/no^2^) | -0.03 | 0.03 | -0.84 | 0.402 | 0.00 |
| …is elderly (>60 years) or has underlying health condition (yes/no^2^) | 0.10 | 0.03 | 2.93 | 0.003 | 0.01^a^ |
| …lives in a senior’s residence (yes/no^2^) | 0.03 | 0.04 | 0.59 | 0.554 | 0.00 |
| …lives in a long-term care home (yes/no^2^) | -0.05 | 0.05 | -1.12 | 0.264 | 0.00 |
| …has had COVID-19 and their outcome |  |  |  |  |  |
| With mild symptoms | -0.14 | 0.05 | -2.74 | 0.006 | 0.00 |
| Moderate-to-severe without hospitalization | -0.09 | 0.05 | -1.73 | 0.084 | 0.00 |
| Moderate-to-severe with hospitalization | -0.05 | 0.07 | -0.79 | 0.432 | 0.00 |
| Required admission to an intensive care unit | -0.09 | 0.10 | -0.83 | 0.409 | 0.00 |
| Deceased | -0.13 | 0.07 | -1.84 | 0.066 | 0.00 |
| Does not know anyone affected^2^ | - | - | - | - | - |
| Prior laboratory testing for COVID-19 |  |  |  |  |  |
| Tested + | 0.01 | 0.10 | 0.11 | 0.911 | 0.00 |
| Tested - | 0.06 | 0.04 | 1.33 | 0.184 | 0.00 |
| Tested and pending | 0.02 | 0.13 | 0.17 | 0.868 | 0.00 |
| Never tested^2^ | - | - | - | - | - |
| COVID-19 health risk factors (total score^4^) | 0.03 | 0.01 | 1.93 | 0.053 | 0.00 |
| Believing one is infected with COVID-19 | -0.02 | 0.01 | -2.90 | 0.004 | 0.00 |
| Believing one need testing for COVID-19 | 0.01 | 0.01 | 1.08 | 0.280 | 0.00 |
| Reduction in income due to COVID-19 | 0.01 | 0.01 | 1.21 | 0.225 | 0.00 |
| Negative impact of social distancing on mental health | 0.02 | 0.02 | 1.21 | 0.225 | 0.00 |
| Negative impact of COVID-19 on mental health | -0.03 | 0.02 | -2.13 | 0.033 | 0.00 |
| Source of health information |  |  |  |  |  |
| Friends or family | -0.22 | 0.08 | -2.78 | 0.005 | 0.00 |
| Doctor | -0.05 | 0.04 | -1.30 | 0.195 | 0.00 |
| Social media | -0.12 | 0.07 | -1.65 | 0.099 | 0.00 |
| Internet | -0.04 | 0.04 | -0.98 | 0.328 | 0.00 |
| Radio/Podcast | -0.06 | 0.09 | -0.64 | 0.521 | 0.00 |
| Newspaper | -0.06 | 0.07 | -0.85 | 0.395 | 0.00 |
| Magazines | -0.27 | 0.29 | -0.95 | 0.343 | 0.00 |
| Television^2^ | - | - | - | - | - |
| Origin of COVID-19 |  |  |  |  |  |
| It was developed intentionally in a lab | -0.03 | 0.04 | -0.65 | 0.518 | 0.00 |
| It was made accidentally in a lab | -0.02 | 0.05 | -0.28 | 0.780 | 0.00 |
| It doesn't really exist | -0.60 | 0.13 | -4.53 | 0.000 | 0.01^a^ |
| It came about naturally likely from animals to humans^2^ | - | - | - | - | - |
| CTGO, trust in government’s management of COVID-19 | 0.00 | 0.00 | 0.72 | 0.472 | 0.00 |

CTGO, Citizen Trust in Government Organizations’ Scale.

^1^Total adjusted R^2^: 0.33; ^2^Reference variable.

^3^Total score was derived from adding scores for the degree of satisfaction with personal relationships and support from friends.

^4^One point was assigned for each health risk factor (i.e., heart disease, hypertension, lung disease, diabetes, cancer, chronic kidney disease, obesity, and weakened immune system) to derive a total health risk factor score for COVID-19.

^a^Small effect (η^2^=0.01); ^b^Medium effect (η^2^=0.06); ^c^Large effect (η^2^=0.14); *p<0.01 (0.05/3 univariate models)

**Supplemental Material 8.** Univariate analysis examining the association between psychological determinants and social distancing adherence in men.^1^

|  | **Beta** | **SE** | **t** | **p-value** | **Partial η^2^** |
| --- | --- | --- | --- | --- | --- |
| RPS, Risk propensity | -0.15 | 0.01 | -11.43 | <0.001* | 0.05^a^ |
| PVD, Germ aversion subscale | 0.14 | 0.02 | 8.75 | <0.001* | 0.03^a^ |
| PVD, Perceived infectability subscale | 0.02 | 0.02 | 1.27 | 0.206 | 0.00 |
| MISS, Suggestibility | 0.00 | 0.00 | -1.73 | 0.083 | 0.00 |
| DRI, Religiosity/spirituality subscale | 0.00 | 0.00 | -0.47 | 0.639 | 0.00 |
| TIPI, Extraversion | -0.02 | 0.01 | -1.73 | 0.085 | 0.00 |
| TIPI, Agreeableness | 0.02 | 0.02 | 1.08 | 0.279 | 0.00 |
| TIPI, Conscientiousness | 0.01 | 0.01 | 0.66 | 0.511 | 0.00 |
| TIPI, Emotional stability | 0.01 | 0.02 | 0.70 | 0.482 | 0.00 |
| TIPI, Openness to experience | 0.06 | 0.01 | 4.43 | <0.001* | 0.01^a^ |
| VAX, total score^2^ | -0.10 | 0.02 | -6.67 | <0.001* | 0.02^a^ |
| HCAM, Holistic health subscale^3^ | -0.01 | 0.00 | -3.05 | 0.002* | 0.00 |
| HCAM, Complementary and alternative medicine subscale^3^ | 0.01 | 0.00 | 3.16 | 0.002* | 0.00 |
| LOC, Internal | 0.01 | 0.01 | 2.80 | 0.005* | 0.00 |
| LOC, Chance | 0.01 | 0.01 | 0.99 | 0.324 | 0.00 |
| LOC, Powerful others | 0.01 | 0.01 | 1.24 | 0.215 | 0.00 |
| GTS, General trust | 0.13 | 0.02 | 6.10 | <0.001* | 0.02^a^ |
| ABI, Attitude towards authority | 0.00 | 0.00 | 0.56 | 0.574 | 0.00 |
| PANAS, Positive affect score | 0.01 | 0.00 | 3.17 | 0.002* | 0.00 |
| PANAS, Negative affect score | 0.00 | 0.00 | -1.32 | 0.186 | 0.00 |
| ECR, Attachment anxiety subscale | 0.00 | 0.00 | 0.94 | 0.349 | 0.00 |
| ECR, Attachment avoidance subscale | 0.01 | 0.00 | 3.45 | 0.001* | 0.01^a^ |

RPS, Risk Propensity Scale; PVD, Perceived Vulnerability to Disease Questionnaire; MISS, Multidimensional Iowa Suggestibility Scale; DRI, Duke Religion/Spirituality Index; TIPI, Ten-Item Personality Inventory; HCAM, Holistic Complementary and Alternative Medicine Questionnaire; LOC, Brief Locus-of-Control Scale; GTS, General Trust Scale; ABI, Authority Behavior Index; PANAS, Positive and Negative Affect Schedule; ECR, Experiences in Close Relationships Scale.

^1^Total adjusted R^2^: 0.26

^2^Higher scores represent anti-vaccination attitudes.

^3^Higher scores represent a more negative attitude toward holistic complementary and alternative medicine.

^a^Small effect (η^2^=0.01); ^b^Medium effect (η^2^=0.06); ^c^Large effect (η^2^=0.14); *p<0.01 (0.05/3 univariate models)

**Supplemental Material 9.** Univariate analysis examining the association between sociodemographic determinants and social distancing adherence in women.^1^

|  | **Beta** | **SE** | **t** | **p-value** | **Partial η^2^** |
| --- | --- | --- | --- | --- | --- |
| Age | 0.01 | 0.00 | 9.16 | <0.001* | 0.03^a^ |
| Race |  |  |  |  |  |
| Indigenous | 0.01 | 0.14 | 0.05 | 0.964 | 0.00 |
| Black | -0.15 | 0.06 | -2.32 | 0.020 | 0.00 |
| East Asian | 0.16 | 0.05 | 3.57 | <0.001* | 0.01^a^ |
| Latinx | 0.13 | 0.06 | 2.40 | 0.017 | 0.00 |
| South Asian | 0.04 | 0.09 | 0.46 | 0.644 | 0.00 |
| Other | -0.03 | 0.05 | -0.50 | 0.619 | 0.00 |
| White^2^ | - | - | - | - | - |
| Education (years) | 0.01 | 0.00 | 1.63 | 0.103 | 0.00 |
| Region of residence |  |  |  |  |  |
| Canada | 0.08 | 0.03 | 2.58 | 0.010 | 0.00 |
| Florida/Texas | 0.01 | 0.04 | 0.14 | 0.887 | 0.00 |
| New York/California^2^ | - | - | - | - | - |
| Religion (yes/no^2^) | 0.07 | 0.03 | 2.39 | 0.017 | 0.00 |
| Population density |  |  |  |  |  |
| 1,000 or less | -0.13 | 0.08 | -1.56 | 0.119 | 0.00 |
| 1,000 to 29,999 | -0.06 | 0.05 | -1.29 | 0.196 | 0.00 |
| 30,000 to 99,999 | -0.05 | 0.04 | -1.24 | 0.215 | 0.00 |
| 100,000 or more^2^ | - | - | - | - | - |
| Political affiliation |  |  |  |  |  |
| Communism left wing or socialism | 0.15 | 0.06 | 2.35 | 0.019 | 0.00 |
| Liberal | 0.14 | 0.03 | 4.37 | <0.001* | 0.01^a^ |
| Center^2^ | - | - | - | - | - |
| Conservative | -0.16 | 0.04 | -4.48 | <0.001* | 0.01^a^ |
| Fascism right wing or authoritarianism | -0.06 | 0.13 | -0.48 | 0.633 | 0.00 |
| Healthcare worker status (yes/no^2^) | -0.02 | 0.04 | -0.47 | 0.641 | 0.00 |
| Employment status |  |  |  |  |  |
| Unemployed | -0.02 | 0.04 | -0.37 | 0.710 | 0.00 |
| Employed^2^ | - | - | - | - | - |
| Student | 0.10 | 0.06 | 1.61 | 0.108 | 0.00 |
| Retired | 0.05 | 0.05 | 1.03 | 0.303 | 0.00 |
| Dwelling |  |  |  |  |  |
| House with a backyard^2^ | - | - | - | - | - |
| House without a backyard | -0.09 | 0.08 | -1.11 | 0.269 | 0.00 |
| Apartment‎/condominium‎/loft with no or small private outdoor space | -0.05 | 0.03 | -1.47 | 0.142 | 0.00 |
| Apartment‎/condominium‎/loft with large outdoor space | 0.02 | 0.05 | 0.34 | 0.738 | 0.00 |
| Senior's residence | -0.17 | 0.20 | -0.87 | 0.383 | 0.00 |
| Long-term facility‎ or‎ nursing home | -0.38 | 0.47 | -0.82 | 0.412 | 0.00 |
| Household income |  |  |  |  |  |
| Less than $20,000 | -0.14 | 0.06 | -2.43 | 0.015 | 0.00 |
| $20,000-$59,999 | -0.04 | 0.04 | -1.09 | 0.277 | 0.00 |
| $60,000-$99,999^2^ | - | - | - | - | - |
| $100,000-$139,999 | -0.12 | 0.04 | -2.66 | 0.008* | 0.00 |
| $140,000 or more | -0.01 | 0.04 | -0.28 | 0.778 | 0.00 |
| Marital status (single/married^2^) | 0.01 | 0.03 | 0.20 | 0.838 | 0.00 |
| Number of persons in a household | 0.05 | 0.01 | 3.78 | <0.001* | 0.01^a^ |
| Substance use in the past week |  |  |  |  |  |
| Alcohol use (yes/no^2^) | -0.05 | 0.03 | -1.58 | 0.114 | 0.00 |
| Cigarette use (yes/no^2^) | 0.08 | 0.05 | 1.73 | 0.085 | 0.00 |
| Electronic cigarette use (yes/no^2^) | -0.20 | 0.06 | -3.42 | 0.001* | 0.01^a^ |
| Cannabis use (yes/no^2^) | -0.02 | 0.04 | -0.37 | 0.712 | 0.00 |

^1^Total adjusted R^2^: 0.11; ^2^Reference variable.

^a^Small effect (η^2^=0.01); ^b^Medium effect (η^2^=0.06); ^c^Large effect (η^2^=0.14); *p<0.01 (0.05/3 univariate models)

**Supplemental Material 10.** Univariate analysis examining the association between COVID-19 and social distancing determinants and social distancing adherence in women.^1^

|  | **Beta** | **SE** | **t** | **p-value** | **Partial η^2^** |
| --- | --- | --- | --- | --- | --- |
| Degree of social support (total score^3^) | 0.02 | 0.00 | 6.11 | 0.000 | 0.02^a^ |
| Perceived seriousness of COVID-19 | 0.40 | 0.02 | 23.65 | 0.000 | 0.22^c^ |
| Knowing someone personally close who |  |  |  |  |  |
| …is a healthcare worker (yes/no^2^) | -0.01 | 0.03 | -0.21 | 0.832 | 0.00 |
| …is elderly (>60 years) or has underlying health condition (yes/no^2^) | 0.01 | 0.03 | 0.42 | 0.674 | 0.00 |
| …lives in a senior’s residence (yes/no^2^) | -0.02 | 0.04 | -0.42 | 0.675 | 0.00 |
| …lives in a long-term care home (yes/no^2^) | -0.07 | 0.05 | -1.45 | 0.147 | 0.00 |
| …has had COVID-19 and their outcome |  |  |  |  |  |
| With mild symptoms | 0.00 | 0.04 | 0.01 | 0.992 | 0.00 |
| Moderate-to-severe without hospitalization | 0.00 | 0.05 | 0.04 | 0.966 | 0.00 |
| Moderate-to-severe with hospitalization | -0.05 | 0.07 | -0.75 | 0.455 | 0.00 |
| Required admission to an intensive care unit | 0.04 | 0.09 | 0.45 | 0.651 | 0.00 |
| Deceased | 0.01 | 0.07 | 0.14 | 0.890 | 0.00 |
| Does not know anyone affected^2^ | - | - | - | - | - |
| Prior laboratory testing for COVID-19 |  |  |  |  |  |
| Tested + | -0.12 | 0.10 | -1.14 | 0.254 | 0.00 |
| Tested - | -0.04 | 0.04 | -1.10 | 0.273 | 0.00 |
| Tested and pending | 0.21 | 0.19 | 1.09 | 0.277 | 0.00 |
| Never tested^2^ | - | - | - | - | - |
| COVID-19 health risk factors (total score^4^) | -0.01 | 0.01 | -0.92 | 0.357 | 0.00 |
| Believing one is infected with COVID-19 | -0.02 | 0.01 | -2.45 | 0.014 | 0.00 |
| Believing one need testing for COVID-19 | -0.01 | 0.01 | -0.97 | 0.332 | 0.00 |
| Reduction in income due to COVID-19 | 0.02 | 0.01 | 1.76 | 0.078 | 0.00 |
| Negative impact of social distancing on mental health | 0.03 | 0.01 | 2.12 | 0.034 | 0.00 |
| Negative impact of COVID-19 on mental health | -0.05 | 0.01 | -3.71 | 0.000 | 0.01^a^ |
| Source of health information |  |  |  |  |  |
| Friends or family | -0.10 | 0.06 | -1.56 | 0.119 | 0.00 |
| Doctor | -0.02 | 0.04 | -0.53 | 0.596 | 0.00 |
| Social media | -0.18 | 0.06 | -3.06 | 0.002 | 0.01^a^ |
| Internet | -0.05 | 0.04 | -1.27 | 0.204 | 0.00 |
| Radio/Podcast | -0.13 | 0.09 | -1.37 | 0.172 | 0.00 |
| Newspaper | -0.03 | 0.06 | -0.49 | 0.624 | 0.00 |
| Magazines | -0.16 | 0.23 | -0.71 | 0.476 | 0.00 |
| Television^2^ | - | - | - | - | - |
| Origin of COVID-19 |  |  |  |  |  |
| It was developed intentionally in a lab | -0.05 | 0.04 | -1.47 | 0.141 | 0.00 |
| It was made accidentally in a lab | 0.06 | 0.05 | 1.33 | 0.182 | 0.00 |
| It doesn't really exist | -0.16 | 0.14 | -1.18 | 0.240 | 0.00 |
| It came about naturally likely from animals to humans^2^ | - | - | - | - | - |
| CTGO, trust in government’s management of COVID-19 | 0.00 | 0.00 | -1.51 | 0.131 | 0.00 |

CTGO, Citizen Trust in Government Organizations’ Scale.

^1^Total adjusted R^2^: 0.32; ^2^Reference variable.

^3^Total score was derived from adding scores for the degree of satisfaction with personal relationships and support from friends.

^4^One point was assigned for each health risk factor (i.e., heart disease, hypertension, lung disease, diabetes, cancer, chronic kidney disease, obesity, and weakened immune system) to derive a total health risk factor score for COVID-19.

^a^Small effect (η^2^=0.01); ^b^Medium effect (η^2^=0.06); ^c^Large effect (η^2^=0.14); *p<0.01 (0.05/3 univariate models)

**Supplemental Material 11.** Univariate analysis examining the association between psychological determinants and social distancing adherence in women.^1^

|  | **Beta** | **SE** | **t** | **p-value** | **Partial η^2^** |
| --- | --- | --- | --- | --- | --- |
| RPS, Risk propensity | -0.15 | 0.01 | -12.37 | <0.001* | 0.06^b^ |
| PVD, Germ aversion subscale | 0.10 | 0.01 | 7.13 | <0.001* | 0.02^a^ |
| PVD, Perceived infectability subscale | 0.03 | 0.01 | 2.89 | 0.004* | 0.00 |
| MISS, Suggestibility | 0.00 | 0.00 | -2.46 | 0.014 | 0.00 |
| DRI, Religiosity/spirituality subscale | 0.00 | 0.00 | -1.14 | 0.254 | 0.00 |
| TIPI, Extraversion | 0.00 | 0.01 | -0.15 | 0.880 | 0.00 |
| TIPI, Agreeableness | 0.03 | 0.01 | 2.20 | 0.028 | 0.00 |
| TIPI, Conscientiousness | 0.01 | 0.01 | 0.85 | 0.394 | 0.00 |
| TIPI, Emotional stability | 0.01 | 0.01 | 0.89 | 0.374 | 0.00 |
| TIPI, Openness to experience | 0.06 | 0.01 | 5.03 | <0.001* | 0.01^a^ |
| VAX, total score^2^ | -0.07 | 0.01 | -5.11 | <0.001* | 0.01^a^ |
| HCAM, Holistic health subscale^3^ | -0.01 | 0.00 | -4.29 | <0.001* | 0.01^a^ |
| HCAM, Complementary and alternative medicine subscale^3^ | 0.00 | 0.00 | 0.26 | 0.797 | 0.00 |
| LOC, Internal | 0.02 | 0.00 | 4.28 | <0.001* | 0.01^a^ |
| LOC, Chance | 0.01 | 0.00 | 2.46 | 0.014 | 0.00 |
| LOC, Powerful others | 0.00 | 0.00 | 0.40 | 0.692 | 0.00 |
| GTS, General trust | 0.07 | 0.02 | 3.71 | <0.001* | 0.01^a^ |
| ABI, Attitude towards authority | 0.00 | 0.00 | 0.45 | 0.654 | 0.00 |
| PANAS, Positive affect score | 0.00 | 0.00 | 1.74 | 0.083 | 0.00 |
| PANAS, Negative affect score | 0.00 | 0.00 | 0.06 | 0.950 | 0.00 |
| ECR, Attachment anxiety subscale | 0.00 | 0.00 | -0.97 | 0.334 | 0.00 |
| ECR, Attachment avoidance subscale | 0.00 | 0.00 | 2.05 | 0.040 | 0.00 |

RPS, Risk Propensity Scale; PVD, Perceived Vulnerability to Disease Questionnaire; MISS, Multidimensional Iowa Suggestibility Scale; DRI, Duke Religion/Spirituality Index; TIPI, Ten-Item Personality Inventory; HCAM, Holistic Complementary and Alternative Medicine Questionnaire; LOC, Brief Locus-of-Control Scale; GTS, General Trust Scale; ABI, Authority Behavior Index; PANAS, Positive and Negative Affect Schedule; ECR, Experiences in Close Relationships Scale.

^1^Total adjusted R^2^: 0.24

^2^Higher scores represent anti-vaccination attitudes.

^3^Higher scores represent a more negative attitude toward holistic complementary and alternative medicine.

^a^Small effect (η^2^=0.01); ^b^Medium effect (η^2^=0.06); ^c^Large effect (η^2^=0.14); *p<0.01 (0.05/3 univariate models)

**Supplemental Material 12.** Univariate analysis examining the association between sociodemographic determinants and social distancing adherence in participants 60 years of age or older.^1^

|  | **Beta** | **SE** | **t** | **p-value** | **Partial η^2^** |
| --- | --- | --- | --- | --- | --- |
| Age | 0.01 | 0.00 | 2.80 | 0.005* | 0.01^a^ |
| Gender (man/woman^2^) | -0.17 | 0.03 | -5.14 | <0.001* | 0.02^a^ |
| Race |  |  |  |  |  |
| Indigenous | 0.03 | 0.18 | 0.14 | 0.888 | 0.00 |
| Black | 0.01 | 0.10 | 0.15 | 0.884 | 0.00 |
| East Asian | 0.10 | 0.08 | 1.18 | 0.237 | 0.00 |
| Latinx | 0.09 | 0.08 | 1.05 | 0.294 | 0.00 |
| South Asian | 0.25 | 0.16 | 1.51 | 0.132 | 0.00 |
| Other | 0.06 | 0.08 | 0.70 | 0.485 | 0.00 |
| White^2^ | - | - | - | - | - |
| Education (years) | 0.01 | 0.01 | 1.48 | 0.140 | 0.00 |
| Region of residence |  |  |  |  |  |
| Canada | 0.10 | 0.04 | 2.54 | 0.011 | 0.00 |
| Florida/Texas | 0.04 | 0.04 | 0.99 | 0.323 | 0.00 |
| New York/California^2^ | - | - | - | - | - |
| Religion (yes/no^2^) | 0.05 | 0.04 | 1.28 | 0.202 | 0.00 |
| Population density |  |  |  |  |  |
| 1,000 or less | -0.04 | 0.09 | -0.39 | 0.693 | 0.00 |
| 1,000 to 29,999 | 0.00 | 0.06 | 0.00 | 0.998 | 0.00 |
| 30,000 to 99,999 | 0.06 | 0.05 | 1.28 | 0.201 | 0.00 |
| 100,000 or more^2^ | - | - | - | - | - |
| Political affiliation |  |  |  |  |  |
| Communism left wing or socialism | 0.09 | 0.09 | 1.08 | 0.281 | 0.00 |
| Liberal | 0.09 | 0.04 | 2.35 | 0.019 | 0.00 |
| Center^2^ | - | - | - | - | - |
| Conservative | -0.17 | 0.04 | -4.41 | <0.001* | 0.01^a^ |
| Fascism right wing or authoritarianism | -0.74 | 0.30 | -2.50 | 0.013 | 0.00 |
| Healthcare worker status (yes/no^2^) | 0.00 | 0.06 | 0.01 | 0.990 | 0.00 |
| Employment status |  |  |  |  |  |
| Unemployed | -0.01 | 0.08 | -0.12 | 0.901 | 0.00 |
| Employed^2^ | - | - | - | - | - |
| Student | 0.04 | 0.58 | 0.08 | 0.940 | 0.00 |
| Retired | 0.03 | 0.04 | 0.69 | 0.489 | 0.00 |
| Dwelling |  |  |  |  |  |
| House with a backyard^2^ | - | - | - | - | - |
| House without a backyard | 0.10 | 0.12 | 0.81 | 0.420 | 0.00 |
| Apartment‎/condominium‎/loft with no or small private outdoor space | -0.06 | 0.04 | -1.59 | 0.112 | 0.00 |
| Apartment‎/condominium‎/loft with large outdoor space | -0.01 | 0.06 | -0.12 | 0.907 | 0.00 |
| Senior's residence | 0.02 | 0.17 | 0.15 | 0.885 | 0.00 |
| Long-term facility‎ or‎ nursing home | 0.47 | 0.59 | 0.80 | 0.427 | 0.00 |
| Household income |  |  |  |  |  |
| Less than $20,000 | 0.04 | 0.08 | 0.47 | 0.638 | 0.00 |
| $20,000-$59,999 | -0.04 | 0.04 | -0.90 | 0.370 | 0.00 |
| $60,000-$99,999^2^ | - | - | - | - | - |
| $100,000-$139,999 | -0.05 | 0.05 | -0.91 | 0.362 | 0.00 |
| $140,000 or more | -0.07 | 0.05 | -1.56 | 0.120 | 0.00 |
| Marital status (single/married^2^) | -0.03 | 0.04 | -0.70 | 0.484 | 0.00 |
| Number of persons in a household | 0.00 | 0.02 | 0.21 | 0.838 | 0.00 |
| Substance use in the past week |  |  |  |  |  |
| Alcohol use (yes/no^2^) | -0.06 | 0.03 | -1.93 | 0.054 | 0.00 |
| Cigarette use (yes/no^2^) | -0.05 | 0.05 | -0.89 | 0.373 | 0.00 |
| Electronic cigarette use (yes/no^2^) | -0.15 | 0.11 | -1.47 | 0.142 | 0.00 |
| Cannabis use (yes/no^2^) | 0.03 | 0.05 | 0.52 | 0.605 | 0.00 |

^1^Total adjusted R^2^: 0.06; ^2^Reference variable.

^a^Small effect (η^2^=0.01); ^b^Medium effect (η^2^=0.06); ^c^Large effect (η^2^=0.14); *p<0.01 (0.05/3 univariate models)

**Supplemental Material 13.** Univariate analysis examining the association between COVID-19 and social distancing determinants and social distancing adherence in participants 60 years of age or older.^1^

|  | **Beta** | **SE** | **t** | **p-value** | **Partial η^2^** |
| --- | --- | --- | --- | --- | --- |
| Degree of social support (total score^3^) | 0.01 | 0.01 | 2.23 | 0.026 | 0.00 |
| Perceived seriousness of COVID-19 | 0.41 | 0.02 | 16.98 | <0.001* | 0.20^c^ |
| Knowing someone personally close who |  |  |  |  |  |
| …is a healthcare worker (yes/no^2^) | 0.01 | 0.04 | 0.40 | 0.693 | 0.00 |
| …is elderly (>60 years) or has underlying health condition (yes/no^2^) | 0.05 | 0.04 | 1.18 | 0.237 | 0.00 |
| …lives in a senior’s residence (yes/no^2^) | -0.02 | 0.05 | -0.38 | 0.705 | 0.00 |
| …lives in a long-term care home (yes/no^2^) | -0.08 | 0.06 | -1.39 | 0.165 | 0.00 |
| …has had COVID-19 and their outcome |  |  |  |  |  |
| With mild symptoms | -0.09 | 0.06 | -1.62 | 0.106 | 0.00 |
| Moderate-to-severe without hospitalization | -0.08 | 0.06 | -1.30 | 0.193 | 0.00 |
| Moderate-to-severe with hospitalization | 0.07 | 0.09 | 0.79 | 0.431 | 0.00 |
| Required admission to an intensive care unit | -0.11 | 0.15 | -0.70 | 0.486 | 0.00 |
| Deceased | -0.02 | 0.08 | -0.24 | 0.814 | 0.00 |
| Does not know anyone affected^2^ | - | - | - | - | - |
| Prior laboratory testing for COVID-19 |  |  |  |  |  |
| Tested + | -0.01 | 0.32 | -0.04 | 0.971 | 0.00 |
| Tested - | 0.06 | 0.05 | 1.24 | 0.215 | 0.00 |
| Tested and pending | -0.04 | 0.17 | -0.23 | 0.821 | 0.00 |
| Never tested^2^ | - | - | - | - | - |
| COVID-19 health risk factors (total score^4^) | 0.00 | 0.02 | 0.21 | 0.835 | 0.00 |
| Believing one is infected with COVID-19 | 0.01 | 0.01 | 0.49 | 0.624 | 0.00 |
| Believing one need testing for COVID-19 | 0.00 | 0.01 | -0.47 | 0.637 | 0.00 |
| Reduction in income due to COVID-19 | 0.01 | 0.01 | 0.69 | 0.491 | 0.00 |
| Negative impact of social distancing on mental health | -0.01 | 0.02 | -0.53 | 0.594 | 0.00 |
| Negative impact of COVID-19 on mental health | 0.00 | 0.02 | 0.19 | 0.850 | 0.00 |
| Source of health information |  |  |  |  |  |
| Friends or family | -0.08 | 0.13 | -0.64 | 0.521 | 0.00 |
| Doctor | 0.00 | 0.04 | -0.01 | 0.996 | 0.00 |
| Social media | -0.04 | 0.18 | -0.25 | 0.803 | 0.00 |
| Internet | 0.05 | 0.05 | 0.98 | 0.330 | 0.00 |
| Radio/Podcast | -0.08 | 0.11 | -0.68 | 0.499 | 0.00 |
| Newspaper | 0.00 | 0.06 | -0.01 | 0.993 | 0.00 |
| Magazines | 0.18 | 0.40 | 0.45 | 0.650 | 0.00 |
| Television^2^ | - | - | - | - | - |
| Origin of COVID-19 |  |  |  |  |  |
| It was developed intentionally in a lab | 0.01 | 0.05 | 0.23 | 0.820 | 0.00 |
| It was made accidentally in a lab | 0.05 | 0.05 | 1.00 | 0.319 | 0.00 |
| It doesn't really exist | 0.06 | 0.22 | 0.27 | 0.791 | 0.00 |
| It came about naturally likely from animals to humans^2^ | - | - | - | - | - |
| CTGO, trust in government’s management of COVID-19 | 0.00 | 0.00 | -1.09 | 0.274 | 0.00 |

CTGO, Citizen Trust in Government Organizations’ Scale.

^1^Total adjusted R^2^: 0.23; ^2^Reference variable.

^3^Total score was derived from adding scores for the degree of satisfaction with personal relationships and support from friends.

^4^One point was assigned for each health risk factor (i.e., heart disease, hypertension, lung disease, diabetes, cancer, chronic kidney disease, obesity, and weakened immune system) to derive a total health risk factor score for COVID-19.

^a^Small effect (η^2^=0.01); ^b^Medium effect (η^2^=0.06); ^c^Large effect (η^2^=0.14); *p<0.01 (0.05/3 univariate models)

**Supplemental Material 14.** Univariate analysis examining the association between psychological determinants and social distancing adherence in participants 60 years of age or older.^1^

|  | **Beta** | **SE** | **t** | **p-value** | **Partial η^2^** |
| --- | --- | --- | --- | --- | --- |
| RPS, Risk propensity | -0.14 | 0.01 | -10.26 | <0.001* | 0.07^b^ |
| PVD, Germ aversion subscale | 0.07 | 0.02 | 4.43 | <0.001* | 0.01^a^ |
| PVD, Perceived infectability subscale | 0.05 | 0.01 | 3.29 | 0.001* | 0.01^a^ |
| MISS, Suggestibility | 0.00 | 0.00 | -0.77 | 0.444 | 0.00 |
| DRI, Religiosity/spirituality subscale | 0.00 | 0.00 | -0.29 | 0.774 | 0.00 |
| TIPI, Extraversion | -0.01 | 0.01 | -0.78 | 0.434 | 0.00 |
| TIPI, Agreeableness | 0.07 | 0.02 | 4.51 | <0.001* | 0.01 |
| TIPI, Conscientiousness | 0.02 | 0.02 | 1.18 | 0.237 | 0.00 |
| TIPI, Emotional stability | -0.02 | 0.02 | -1.31 | 0.191 | 0.00 |
| TIPI, Openness to experience | 0.07 | 0.01 | 4.84 | <0.001* | 0.02^a^ |
| VAX, total score^2^ | -0.05 | 0.02 | -2.82 | 0.005* | 0.01^a^ |
| HCAM, Holistic health subscale^3^ | -0.01 | 0.00 | -1.10 | 0.272 | 0.00 |
| HCAM, Complementary and alternative medicine subscale^3^ | 0.00 | 0.00 | 0.68 | 0.498 | 0.00 |
| LOC, Internal | 0.00 | 0.01 | 0.79 | 0.430 | 0.00 |
| LOC, Chance | 0.00 | 0.01 | 0.27 | 0.789 | 0.00 |
| LOC, Powerful others | 0.00 | 0.00 | 0.04 | 0.972 | 0.00 |
| GTS, General trust | 0.10 | 0.02 | 4.38 | <0.001* | 0.01^a^ |
| ABI, Attitude towards authority | 0.00 | 0.00 | -0.85 | 0.396 | 0.00 |
| PANAS, Positive affect score | 0.00 | 0.00 | 0.56 | 0.578 | 0.00 |
| PANAS, Negative affect score | 0.00 | 0.00 | -0.53 | 0.593 | 0.00 |
| ECR, Attachment anxiety subscale | 0.00 | 0.00 | -0.28 | 0.779 | 0.00 |
| ECR, Attachment avoidance subscale | 0.01 | 0.00 | 3.17 | 0.002* | 0.01^a^ |

RPS, Risk Propensity Scale; PVD, Perceived Vulnerability to Disease Questionnaire; MISS, Multidimensional Iowa Suggestibility Scale; DRI, Duke Religion/Spirituality Index; TIPI, Ten-Item Personality Inventory; HCAM, Holistic Complementary and Alternative Medicine Questionnaire; LOC, Brief Locus-of-Control Scale; GTS, General Trust Scale; ABI, Authority Behavior Index; PANAS, Positive and Negative Affect Schedule; ECR, Experiences in Close Relationships Scale.

^1^Total adjusted R^2^: 0.20

^2^Higher scores represent anti-vaccination attitudes.

^3^Higher scores represent a more negative attitude toward holistic complementary and alternative medicine.

^a^Small effect (η^2^=0.01); ^b^Medium effect (η^2^=0.06); ^c^Large effect (η^2^=0.14); *p<0.01 (0.05/3 univariate mod


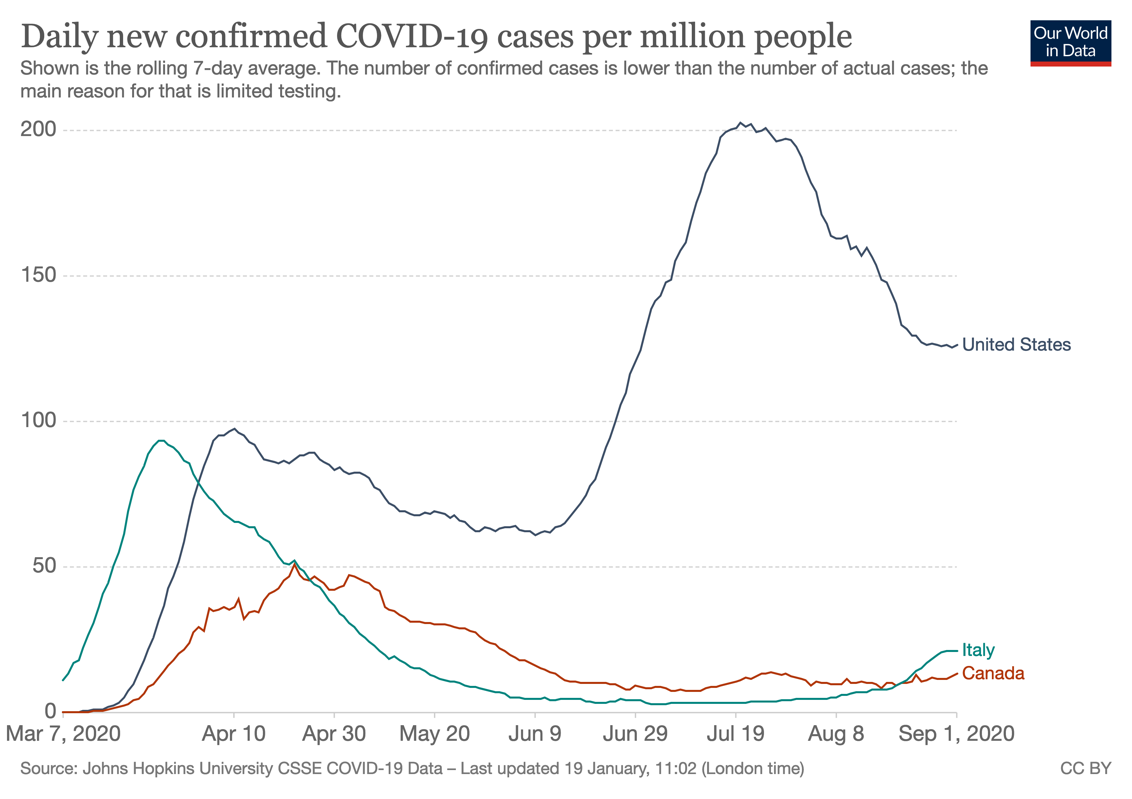


**Supplemental Figure 1.** Weekly number of cases per million people prior to the second wave. Comparing the United States and Canada, who enacted social distancing restrictions relatively late and early, respectively, to Italy who were unable to or chose not to adopt social distancing restrictions early. Source: COVID-19 Data Repository by the Center for Systems Science and Engineering at Johns Hopkins University (17)via Our World in Data.

*

*

*

**Supplemental Figure 2**. Mean social distancing adherence scores across political affiliations. Error bars represent $\pm$1 standard error. *Bonferroni-corrected p-value <0.05 with center as the reference group.

**References**

1. Rigby K. An Authority Behavior Inventory. *Journal of Personality Assessment* (1987) 51:615–625. doi: 10.1207/s15327752jpa5104_14

2. Centers for Disease Control and Prevention. How to Protect Yourself & Others. *Centers for Disease Control and Prevention* (2020) https://www.cdc.gov/coronavirus/2019-ncov/prevent-getting-sick/prevention.html [Accessed January 21, 2021]

3. Public Health Agency of Canada. Coronavirus disease (COVID-19): Prevention and risks. (2020) https://www.canada.ca/en/public-health/services/diseases/2019-novel-coronavirus-infection/prevention-risks.html [Accessed May 3, 2020]

4. World Health Organization (WHO). Coronavirus disease (COVID-19) advice for the public. (2021) https://www.who.int/emergencies/diseases/novel-coronavirus-2019/advice-for-public [Accessed January 21, 2021]

5. Grimmelikhuijsen S, Knies E. Validating a scale for citizen trust in government organizations: *International Review of Administrative Sciences* (2015) doi: 10.1177/0020852315585950

6. Meertens RM, Lion R. Measuring an individual’s tendency to take risks: The risk propensity scale. *Journal of Applied Social Psychology* (2008) 38:1506–1520. doi: 10.1111/j.1559-1816.2008.00357.x

7. Duncan LA, Schaller M, Park JH. Perceived vulnerability to disease: Development and validation of a 15-item self-report instrument. *Personality and Individual Differences* (2009) 47:541–546. doi: 10.1016/j.paid.2009.05.001

8. Kotov R.I., Bellman S.B., Watson D.B. Multidimensional Iowa Suggestibility Scale (MISS) Brief Manual. (2004). https://dspace.sunyconnect.suny.edu/handle/1951/60894

9. Koenig H, Parkerson GR, Meador KG. Religion index for psychiatric research. *Am J Psychiatry* (1997) 154:885–886. doi: 10.1176/ajp.154.6.885b

10. Gosling SD, Rentfrow PJ, Swann WB. A very brief measure of the Big-Five personality domains. *Journal of Research in Personality* (2003) 37:504–528. doi: 10.1016/S0092-6566(03)00046-1

11. Martin LR, Petrie KJ. Understanding the Dimensions of Anti-Vaccination Attitudes: the Vaccination Attitudes Examination (VAX) Scale. *Ann Behav Med* (2017) 51:652–660. doi: 10.1007/s12160-017-9888-y

12. Hyland ME, Lewith GT, Westoby C. Developing a measure of attitudes: the holistic complementary and alternative medicine questionnaire. *Complement Ther Med* (2003) 11:33–38. doi: 10.1016/s0965-2299(02)00113-9

13. Sapp SG, Harrod WJ. Reliability and validity of a brief version of Levenson’s Locus of Control Scale. *Psychological Reports* (1993) 72:539–550. doi: 10.2466/pr0.1993.72.2.539

14. Yamagishi T, Yamagishi M. Trust and commitment in the United States and Japan. *Motiv Emot* (1994) 18:129–166. doi: 10.1007/BF02249397

15. Crawford JR, Henry JD. The positive and negative affect schedule (PANAS): construct validity, measurement properties and normative data in a large non-clinical sample. *The British journal of clinical psychology / the British Psychological Society* (2004) 43:245–65. doi: 10.1348/0144665031752934

16. Brennan KA, Clark CL, Shaver PR. Self-report measurement of adult attachment: An integrative overview. (1998) doi: null

17. Dong E, Du H, Gardner L. An interactive web-based dashboard to track COVID-19 in real time. *The Lancet Infectious Diseases* (2020) 20:533–534. doi: 10.1016/S1473-3099(20)30120-1
